# Management of Children with Acute Asthma Attack: A RAND/UCLA Appropriateness Approach

**DOI:** 10.3390/ijerph182312775

**Published:** 2021-12-03

**Authors:** Valentina Fainardi, Carlo Caffarelli, Barbara Maria Bergamini, Loretta Biserna, Paolo Bottau, Elena Corinaldesi, Arianna Dondi, Martina Fornaro, Battista Guidi, Francesca Lombardi, Maria Sole Magistrali, Elisabetta Marastoni, Alessandra Piccorossi, Maurizio Poloni, Sylvie Tagliati, Francesca Vaienti, Cristina Venturelli, Giampaolo Ricci, Susanna Esposito, on behalf of the Emilia-Romagna Asthma (ERA) Study Group

**Affiliations:** 1 Pediatric Clinic, Department of Medicine and Surgery, University of Parma, 43126 Parma, Italy; valentina.fainardi@unipr.it (V.F.); carlo.caffarelli@unipr.it (C.C.); 2 Paediatric Unit, Department of Medical and Surgical Sciences of Mothers, Children and Adults, University of Modena and Reggio Emilia, 41125 Modena, Italy; barbaramaria.bergamini@unimore.it; 3 Paediatrics and Neonatology Unit, Ravenna Hospital, AUSL Romagna, 48121 Ravenna, Italy; lorettabiserna@libero.it; 4 Paediatrics Unit, Imola Hospital, 40026 Imola, Italy; p.bottau@ausl.imola.bo.it; 5 Paediatric Unit, Carpi Hospital, 41012 Carpi, Italy; elcorinaldesi@yahoo.it; 6 Scientific Institute for Research and Healthcare (IRCCS) Azienda Ospedaliero-Universitaria di Bologna, 40138 Bologna, Italy; arianna.dondi@aosp.bo.it; 7 Paediatrics and Neonatology Unit, Macerata Hospital, ASUR Marche–AV3, 62100 Macerata, Italy; martina.fornaro@sanita.marche.it; 8 Hospital and Territorial Paediatrics Unit, Pavullo Hospital, 41026 Pavullo nel Frignano, Italy; b.guidi@ausl.mo.it; 9 Paediatrics Unit, Maggiore Hospital, 40133 Bologna, Italy; francesca.lombardi@ausl.bologna.it; 10 Paediatrics and Neonatology Unit, Guglielmo da Saliceto Hospital, 29121 Piacenza, Italy; m.magistrali2@ausl.pc.it; 11 Paediatrics Unit, Santa Maria Nuova Hospital, AUSL-IRCCS of Reggio Emilia, 42123 Reggio Emilia, Italy; Elisabetta.Marastoni@ausl.re.it; 12 Paediatrics and Paediatric Intensive Care Unit, Cesena Hospital, AUSL Romagna, 47521 Cesena, Italy; alessandrafaustamarcella.piccorossi@auslromagna.it; 13 Paediatrics Unit, Rimini Hospital, AUSL Romagna, 47921 Rimini, Italy; polonimaurizio@gmail.com; 14 Paediatric Clinic, Ferrara Hospital, 44124 Ferrara, Italy; sylvie.tagliati@unife.it; 15 Paediatrics Unit, G.B. Morgagni—L. Pierantoni Hospital, AUSL Romagna, 47121 Forlì, Italy; francesca.vaienti@auslromagna.it; 16 Paediatrics Unit, Sassuolo Hospital, 41049 Sassuolo, Italy; cri.venturelli@ospedalesassuolo.it; 17 Alma Mater Studiorum, Department of Medical and Surgical Sciences, University of Bologna, 40138 Bologna, Italy; giampaolo.ricci@unibo.it

**Keywords:** asthma, good clinical practices, respiratory exacerbation, spirometry, teleconsultation

## Abstract

Bronchial asthma is the most frequent chronic disease in children and affects up to 20% of the pediatric population, depending on the geographical area. Asthma symptoms vary over time and in intensity, and acute asthma attack can resolve spontaneously or in response to therapy. The aim of this project was to define the care pathway for pediatric patients who come to the primary care pediatrician or Emergency Room with acute asthmatic access. The project was developed in the awareness that for the management of these patients, broad coordination of interventions in the pre-hospital phase and the promotion of timely and appropriate assistance modalities with the involvement of all health professionals involved are important. Through the application of the RAND method, which obliges to discuss the statements derived from the guidelines, there was a clear increase in the concordance in the behavior on the management of acute asthma between primary care pediatricians and hospital pediatricians. The RAND method was found to be useful for the selection of good practices forming the basis of an evidence-based approach, and the results obtained form the basis for further interventions that allow optimizing the care of the child with acute asthma attack at the family and pediatric level. An important point of union between the primary care pediatrician and the specialist hospital pediatrician was the need to share spirometric data, also including the use of new technologies such as teleconsultation. Monitoring the progress of asthma through spirometry could allow the pediatrician in the area to intervene early by modifying the maintenance therapy and help the patient to achieve good control of the disease.

## 1. Introduction

Asthma is the most frequent chronic disease of childhood, affecting up to 20% of children depending on the geographical area [1]. It is characterized by chronic airway inflammation and airway hyper-responsiveness in response to triggers that can cause acute symptoms and eventually airway remodeling [2,3]. Asthma causes symptoms such as wheezing, shortness of breath, chest tightness and cough that vary over time in their occurrence, frequency and intensity. People with asthma generally have more than one of these symptoms. The symptoms have these characteristics: they occur variably over time and vary in intensity; they often occur or are worse at night or on waking; they are often triggered by exercise, laughter, allergens or cold air; they often occur with or worsen with viral infections; they are associated with evidence of variable expiratory airflow limitation [3]. According to GINA recommendations [3], asthma severity is assessed retrospectively from the level of treatment required to control symptoms and exacerbations. Asthma severity may change over months and can be classified when the patient has been on controller treatment for several months as: (1) mild, if asthma is well controlled with step 1 or step 2 treatment (as needed inhaled corticosteroids [ICS]-formoterol or low dose ICS or leukotriene receptor antagonists); (2) moderate, if asthma is well controlled with step 3 or step 4 treatment (low or medium dose ICS/long-acting β-agonists [LABA]); (3) severe, if asthma is well controlled with step 5 treatment (high dose ICS/LABA or biologics). Biologic drugs are recommended for patients with exacerbations or poor symptom control despite taking at least high dose ICS/LABA, and who have allergic or eosinophilic biomarkers or need maintenance oral corticosteroids (OCS) [3]. A careful medical history, physical examination, assessment of atopy, comorbidities and lung function are essential to confirm the diagnosis of asthma and exclude other diseases [2,4,5]. Asthma control means the extent to which the effects of asthma can be seen in the patient, or have been reduced or removed by treatment [3]. Asthma control has two domains: symptom control and risk factors for future poor outcomes, particularly flare-ups (exacerbations). The acute attack of asthma is characterized by bronchoconstriction of the bronchial smooth muscles, inflammation and mucus secretion [3]. Symptoms include cough, wheezing, chest tightness, dyspnea and difficulty in carrying out daily activities. These symptoms are associated with difficulty in breathing air out of the lungs due to bronchoconstriction (airway narrowing), airway wall thickening and increased mucus. Some variation in airflow can also occur in people without asthma, but it is greater in untreated asthma [3]. Spirometry often shows variable expiratory airflow limitation. The attack can resolve spontaneously or after appropriate treatment. Severe attacks can manifest with respiratory failure and need prompt medical examination. The acute attack occurs regardless of the different phenotypes (observable clinical characteristics) and endotypes (underlying mechanisms of pathogenesis) of asthma [4]. Prevention of asthma attacks includes avoidance of triggers (allergens, infections, smoke and other pollutants), assessment and treatment of comorbidities (rhinosinusitis, obesity, gastroesophageal reflux, obstructive apnea syndrome, anxiety) [6] and self-management education with a written personalized asthma action plan. Maintenance treatment must be adapted to each patient with the lowest effective dose in order to achieve asthma control and reduce exacerbations and absences from school, allow physical activity and improve quality of life [7,8].

This project aims to review the available evidence on the management of asthma attack in children and develop a shared protocol between hospital and primary care pediatricians to: (a) improve self-management education; (b) describe treatment; (c) define hospital admission criteria; (d) plan post-discharge follow-up.

## 2. Materials and Methods

### 2.1. RAND/UCLA Appropriateness Method

The RAND/UCLA method for assessing appropriateness was used to develop the Consensus document [9]. The RAND/UCLA method involves the assessment by a panel of experts of the appropriateness of a series of diagnostic and therapeutic “procedures” according to the clinical picture of the patient, in situations in which scientific evidence is sub-optimal. According to the RAND method, a procedure is considered “appropriate” if the expected benefits outweigh the expected negative consequences by a sufficiently large margin to justify the procedure, without taking costs into account [10]. Conversely, a procedure is considered “inappropriate” if the risk exceeds the expected benefits. According to the RAND definition, the expert making a judgment of appropriateness/inappropriateness must consider the clinical benefits and not be influenced by economic considerations [11]; therefore, the definition of appropriateness means the assessment of the risk/benefit ratio of a list of diagnostic and therapeutic procedures. The experts answered anonymously to a questionnaire, followed by an open discussion of the survey, and by a possible second-round questionnaire to minimize any disagreement in their answers. Each response was then classified as “appropriate”, “inappropriate” or uncertain”.

### 2.2. Literature Search

Two reviewers (VF and CC) independently searched for experimental studies, reviews, systematic reviews, metanalysis and guidelines using the MEDLINE database from 1 January 2000 to 30 April 2021 (search strategy: [(asthma attack) OR (wheezing) OR (asthma exacerbation) and (management) and (children)]). Only English-language articles were selected. Secondly, the search was completed by a manual review of articles and bibliographies. The selected papers were then provided to the panelists to ensure them an equal and appropriate body of evidence.

### 2.3. Questionnaire Development

The document consisted of 13 clinical scenarios with a total of 91 questions (Supplementary Material S1), developed by the coordinators together with the Heads of the Paediatric Units of Emilia-Romagna and a patients’ association (Respiro Libero, Parma, Italy).

### 2.4. Panel Selection

A multidisciplinary group of 77 specialists in the field of general pediatrics, pediatrics subspecialties (pulmonology and allergy) and a sample of primary care pediatricians (identified in each province on the basis of the number of the pediatric population according to ISTAT 2018 data) was randomly selected among those working in Emilia-Romagna Region, an Italian Region with 4,459,000 inhabitants. All panelists were recruited via phone and email contact and, after acceptance, each one of them was provided with a panel document including the literature review, definitions and instructions and an online questionnaire.

### 2.5. First Round

The experts had to rank the appropriateness of each scenario and indication from ‘1′ to ‘9′ (ranging from extremely not appropriate to extremely appropriate). Grades from ‘1′ to ‘3′ were considered inappropriate, grades from ‘4′ to ‘6′ were considered indeterminate or equivocal, and grades from ‘7′ to ‘9′ were considered appropriate. In assessing each individual indication, each expert referred both to his or her own experience and clinical judgment and to the available scientific evidence. A free space was provided for annotations or comments.

The kick-off meeting of the project was on 10 June 2021, via Teams. The first round of questionnaire responses was carried out “blind” to the judgment of the other panel members (the panel of experts who responded—26 pediatricians who worked in the hospital and 51 primary care pediatricians—were given one month to rank the appropriateness) via the online platform Google forms. Responses to the first round were collected and sent to an independent statistician for analysis of the results.

### 2.6. Data Analysis and Definition of Disagreement/Agreement

Aggregate results were reported as frequencies and means. The mean and disagreement were classified into three levels of appropriateness (appropriate: between ‘7’ and ‘9′, without disagreement; uncertain: between ‘4’ and ‘6’ or any median with disagreement; inappropriate: between ‘1’ and ‘3’ with agreement) (Table 1). Agreement was reached in case of at least 75% of participants ranking within the same level of appropriateness. The data analysis was performed with the STATA^®^ Statistical Software (Release 11 College Station, TX, College Station, TX, USA). The mean value with 95% confidence interval was then calculated. Microsoft Excel^®^ was used for graphic data processing.

### 2.7. Second Round and Consensus Meeting

Participants were asked to re-rank the scenarios in a second round after clarifications, adaptations and refinements of the indications. The difference between the results of the first and second round was discussed in a web meeting on 14 September 2021 where the collective ranking of scenarios and indications were shown (Supplementary Material S2).

## 3. Results

### 3.1. SCENARIO 1. The Action Plan for the Patient in Case of Asthma Attack

The panel ranked four approaches in the case of an asthmatic patient with a mild or moderate asthma attack. Agreement was obtained, both in the first and second round, for recommending the patient to immediately use bronchodilator therapy with short-acting β-agonist (SABA) (96.8% appropriate) while maintaining therapy with ICS (96.8% appropriate). There was a lack of consensus for starting the oral steroid immediately (66.7% inappropriate, 25.4% uncertain) and sending the patient immediately to the Emergency Room (ER; 63.5% inappropriate, 36.5% uncertain).

### 3.2. SCENARIO 2. Evaluation of the Exacerbation in the Emergency Room (ER)

The panel considered ten indications to send the patient with asthma attack to the ER. In the first and second round agreement was obtained for seven cases: SpO_2_ < 92% (98.4% appropriate), the patient is unable to speak (96.8% appropriate) or has altered consciousness (98.4% appropriate), coexistence of comorbidities (90.5% appropriate), medical history of a previous severe asthma attack (95.2% appropriate) and family compliance is poor (95.2% appropriate). Participants also agreed on the administration of SABA rescue therapy if SpO_2_ is <95% before sending the patient to ER (97% appropriate).

### 3.3. SCENARIO 3. Treatment of the Acute Attack

Several options of pharmacological treatment including SABA (six questions), steroids (sixteen questions), ipratropium bromide (three questions), antibiotics (one question), steroids (14 questions) and second-line medications such as leukotriene receptor antagonists, epinephrine, aminophylline and magnesium sulfate (five questions) were presented to the panel.

Participant agreed on the following indications: SABA is the drug of choice (100% appropriate) and can be administered three times every 20 min (100% appropriate); a nebulizer should be preferred to a metered-dose inhaler (MDI) when oxygen is needed (98.4% appropriate); therapy with long-acting beta-2 agonists (LABA) should be suspended when SABA is used more frequently than every 4 h (95.2% appropriate); antibiotics are not a first choice treatment (96.8% appropriate); steroids are not always administered intravenously (88.9% appropriate) but the intravenous route should be preferred when the patient is unable to take them orally (100% appropriate); effect of steroids are evident within 3–4 h (92.1% appropriate); steroid course should be 3–5 days long (87.3% appropriate) and there is no need to always taper them off (85.7% inappropriate) especially if used for less than a week (92.1% appropriate); steroid of choice is prednisone (88.9% appropriate) or dexamethasone (81% appropriate); the oral administration of steroids is equal to intravenous administration (95.2% appropriate); ipratropium bromide has to be considered when the response to SABA is poor (88.9% appropriate) and can be administered every 20 min (81% appropriate); nebulized epinephrine is not indicated for severe asthma attack (87.3% inappropriate) or when the response to first line therapy is poor (90.5% inappropriate); leukotriene receptor antagonists are not indicated when the response to first line therapy is poor (98.4% inappropriate).

Lack of consensus was obtained for: SABA can be administered indifferently using a nebulizer or a MDI with spacer (68.3% appropriate, 22.2% inappropriate) but a nebulizer should be preferred when response to MDI is poor (60.3% appropriate, 25.4% inappropriate); systemic steroids are always needed (42.9% uncertain, 28.6% inappropriate); steroids are always administered orally (73% appropriate) or intravenously in severe cases (68.3% appropriate, 19% inappropriate); steroid of first choice is betamethasone (41.3% inappropriate, 39.7% appropriate); steroid course should be 2–3 days long (65.1% inappropriate, 22.2% appropriate); ipratropium bromide should always be used (63.5% inappropriate, 33.3% uncertain); aminophylline (54% uncertain, 23.8% appropriate) and magnesium sulphate (55.6% uncertain, 28.6% appropriate) can be given when the response to first line therapy is poor; ICS are always needed (49.2% inappropriate, 28.6% uncertain) and should be suspended when oral corticosteroids (OCS) are given (58.7% inappropriate, 38.1% appropriate).

Lack of consensus emerged and was confirmed in the second round on what to do when the response to SABA is poor (27% appropriate, 42.9% uncertain) or if the patient is atopic (47.6% inappropriate, 33.3% uncertain).

### 3.4. SCENARIO 4 and SCENARIO 5. Oxygen Therapy and Types of Ventilation

The panel answered six questions about applying oxygen when oxygen saturation falls below a specified threshold and about the mode of oxygen delivery. Agreement was reached for the SpO_2_ target that has to be considered to start oxygen therapy: SpO_2_ < 95 (81% appropriate) and SpO_2_ < 92% (98.4% appropriate). No consensus was obtained about the need for oxygen in all asthma attacks (50.8% uncertain, 42.9% inappropriate and about the method of oxygen delivery: nasal cannula (54% appropriate, 46% uncertain), Venturi mask (68.3% uncertain, 28.6% appropriate) or mask with reservoir (69.8% uncertain, 25.4% appropriate).

Participants were also questioned about the preferred modality of ventilation in case of failure of standard oxygen therapy and first-line treatment. None of the four questions obtained agreement. In case of failure of first-line treatment and standard oxygen therapy: high flow oxygen (58.7% appropriate, 39.7% uncertain), non-invasive continuous positive airway pressure (CPAP; 44.4% appropriate, 47.6% uncertain) or intubation (52.4% inappropriate, 39.7% uncertain) should be considered and an intensivist should be contacted (73% appropriate).

### 3.5. SCENARIO 6. Intensivist Consultation

Excellent agreement was obtained for almost all the indications for which an intensivist should be consulted: patient unable to speak (85.7% appropriate), altered consciousness (100% appropriate), history of an asthma attack treated in the intensive care unit (ICU; 85.7% appropriate), cyanosis unresponsive to oxygen (96.8% appropriate), persistent tachypnea lasting 3 h followed by bradypnea (100% appropriate), gasping (98.4% appropriate), SpO_2_ < 92% for more than 3 h despite oxygen therapy with reservoir (96.8% appropriate), pO_2_ < 60 mmHg (96.8% appropriate), pCO_2_ > 45 mmHg (98.4% appropriate), need of FiO_2_ > 0.50 (96.8% appropriate), need of ventilator support (100% appropriate), pulmonary barotrauma at chest ultrasound (100% appropriate).

No consensus was reached for considering the pediatric asthma severity score (PASS) >6 an indication to call the intensivist (63.5% appropriate, uncertain 15.9%).

### 3.6. SCENARIO 7 and SCENARIO 8. Chest X-ray and Chest Ultrasound

The panel agreed that during an asthma attack, a chest X-ray should not always be performed (95.2% inappropriate). Uncertainty emerged about the need of a chest X-ray if the patient is febrile (28.6% appropriate, 41.3% uncertain), presents comorbidities (63.5% appropriate, 30.2% uncertain), needs oxygen (49.2% inappropriate, 27% uncertain) or in case of poor response to SABA (39.7% appropriate, 31.7% uncertain) and hospitalization (71.4% inappropriate, 17.5% uncertain).

In the case of suspected pneumothorax, participants agreed that a chest ultrasound should not be performed (76.2% inappropriate), while no consensus was obtained in the case of suspected consolidation (55.6% inappropriate, 30.2% uncertain).

### 3.7. SCENARIO 9 and SCENARIO 10. Arterial Blood Gas Analysis and Blood Tests

The majority of participants agreed that arterial blood gas analysis is not recommended in all patients with asthma attack (“inappropriate” from 41.9% in the first round to 81% in the second round), but that can be considered when SpO_2_ is <92% (85.7% appropriate); however, a consensus was not obtained when considering arterial blood analysis for patients with comorbidities (25.4% appropriate, 34.9% uncertain, 39.7% inappropriate) or needing oxygen therapy (38.1% appropriate, 30.2% uncertain, 31.7% inappropriate).

Almost all experts (98.4%) considered it inappropriate to perform blood tests during an asthma attack. Uncertainty persisted in case of hospitalization of the patient (14.3% appropriate, 36.5% uncertain, 49.2% inappropriate) or in case of fever (17.5% appropriate, 49.2% uncertain, 33.3% inappropriate).

### 3.8. SCENARIO 11. Lung Function Test

After the second round, the panel expressed full consensus about the usefulness of spirometry for patients with a diagnosis of asthma (81% appropriate) and about the usefulness of peak expiratory flow (PEF) to diagnose asthma (76.2% appropriate), to assess patients with a diagnosis of asthma (87.3% appropriate) and to define the severity of the attack (90.5% appropriate). Furthermore, a value of peak expiratory flow (PEF) <50% of best personal value was considered as a sign of severe asthma attack by 95.2% of participants.

Agreement was almost obtained for the statement “spirometry is always useful to diagnose asthma” (74.6% appropriate, 15.9% inappropriate).

### 3.9. SCENARIO 12. Admission to the Hospital

Seven indications for hospital admission were submitted to participants, three of which received full agreement: associated pneumothorax (100% appropriate), need for oxygen supplementation (92.1% appropriate) and poor family compliance (96.8% appropriate). In the second round most participants agreed that hospital admission is not always recommended in case of asthma attack (76.2% inappropriate).

Uncertainty about the need of hospitalization remained when considering patient’s age (<6 years: 57.1% inappropriate, 38.1% uncertain; <1 year: 47.6% appropriate, 31.7% uncertain), or the concomitance of bronchopneumonia (63.5% appropriate, 30.2% uncertain).

### 3.10. SCENARIO 13. Follow-Up after an Asthma Attack

The panel was interviewed with nine options to be considered in the follow-up after an acute asthma attack. Four options reached agreement before and after the second round: specialist consultation is indicated if the patient needed hospital admission (93.7% appropriate) or if the patient presents comorbidities such as bronchiectasis, congenital malformations affecting the respiratory system, prematurity or bronchopulmonary dysplasia (BPD) (98.4% appropriate), a spirometry is recommended if a patient is at least 5 years old (92.1% appropriate), skin prick test for inhaled allergens is always recommended (82% appropriate).

Uncertainty also remained after the second round for: a specialist consultation is always needed after an asthma attack (58.7% appropriate, 25.4% uncertain, 15.9% inappropriate), measurement of fractional exhaled nitric oxide (FeNO) (65.1% appropriate, 34.9% uncertain) and total and specific serum IgE (44.4% uncertain, 55.6% inappropriate) are recommended, skin prick test is indicated only when the child is at least 2 years old (50.8% appropriate, 20.6% uncertain, 28.6% inappropriate), allergy tests are recommended only in case of family history for allergies (9.5% appropriate, 27% uncertain, 63.5% inappropriate).

## 4. Discussion

### 4.1. The Action Plan for the Patient in Case of Asthma Attack

The most recent systematic reviews and international guidelines agreed on the benefit of a written action plan for all patients diagnosed with asthma [3,11,12,13,14,15,16,17,18]. As needed low dose ICS-formoterol or SABA plus a low dose ICS as a reliever are the preferred approaches for a lower risk of exacerbation and hospitalization compared to SABA alone. For patients on maintenance with ICS-formoterol combination, it is recommended to continue the therapy and increase the number of inhalations [19,20,21,22,23,24,25,26,27]. The action plan should help the patient and/or the caregiver to: (a) recognize the symptoms of asthma and the exacerbation; (b) optimize medications; (c) provide instructions about when and how to start OCS; (d) understand when emergency treatment in the hospital is needed [3,11,12,13,14,15,16,17,18]. The severity of asthma attack according to the most recent Italian guidelines is reported in Table 2 [19].

In the second round, the percentage of participants who disagreed about immediately sending the patient to the ER increased from 47.3 to 63.5% without, however, reaching full agreement. This lack of consensus might be ascribed to the heterogeneity of the experts involved, some working in the hospital and some as general pediatricians in primary care.

**Recommendation 1.** In case of a mild/moderate asthma attack, the patient in follow-up for asthma should be invited to take SABA and to continue its maintenance therapy with ICS. In the event of severe attack and/or poor response to SABA within the first hour of treatment, the patient should start oral steroids and go to the hospital.

### 4.2. Evaluation of the Exacerbation in the Emergency Room (ER)

Physical examination and medical history, including previous attacks and comorbidities (Table 3), are the cornerstones to assess the severity of the exacerbation and the need for ER evaluation [3,11,19]. The objective datum of oxygen saturation by pulse oximetry is crucial to support the diagnosis and evaluate asthma attack [23,28,29,30,31,32,33,34,35,36,37,38,39]. When the response to SABA is scarce within the first hour of treatment, the patient should be sent to the ER. The disagreement found about the need for an examination in the ER in case of a patient with allergies can be explained by the fact that some participants may have considered this allergy as a food allergy, which is a risk factor for anaphylaxis. Most cases of asthma in children are caused by inhaled allergies, and allergy, in general, is not considered a risk factor (Table 4).

**Recommendation 2.** A patient with asthma and SpO_2_ < 95% must promptly take SABA and OCS. If the response is poor, the attack is severe (SpO_2_ < 92%, unable to speak, altered consciousness, dyspnea) or risk factors (recent admission to the hospital for asthma, SABA overuse, recent OCS course, coexistent comorbidities, poor adherence to therapy) are present, the patient needs to go to the ER.

### 4.3. Treatment of the Acute Asthma Attack

The first-line therapy to manage asthma attack is SABA every 20 min for the first hour. Steroid therapy should be performed intravenously only in the most serious cases in case of impossibility of oral administration (vomiting, altered state of consciousness) because there is no evidence that intravenous administration of steroids gives more advantages than oral administration. Steroid therapy may include betamethasone, prednisone or dexamethasone and usually continues for a 3–5-day course without the need of tapering off if the steroid is used for a week. Therapy with anticholinergics (ipratropium bromide) should be administered in case of poor response to SABA and can be administered three times every 20 min in addition to SABA.

In the second round, the percentage of participants who considered pMDI with spacer equal to nebulizer increased but not enough to reach the agreement of 75% despite the evidence of its superiority in case of mild and moderate attacks [53,54,55,56,57,58]. Conversely, nebulization represents the ideal route of administration in case of a severe episode and need of oxygen supplementation, as well as in case of poor response to puffs.

Slight discrepancies emerged in modalities, times of application as well as the route of administration of systemic corticosteroids. Their early use in case of acute asthma exacerbation reduces the use of SABA, the risk of hospitalization or relapse of symptoms without an apparent increase in adverse effects and regardless of the route of administration [3,19,59]. For this reason, the oral route should be preferred in children, especially in liquid formulations, while the intravenous (iv) route should be reserved for patients with emetic episodes, too dyspneic to take oral medications or requiring non-invasive ventilation or intubation [3,19,60]. In the second round, there was an increase in the consideration of dexamethasone as a first choice of steroid (from 56.8 to 81%) for its oral tolerability and the possible shorter duration of therapy.

The different experiences of participants, the use in intensive care settings and the scarce evidence reported in literature likely determined the lack of agreement in the use of aminophylline and magnesium sulfate as second-line therapy in case of severe asthma exacerbations. Evidence is still poor about interrupting ICS in the case of OCS course. In addition, some experts of the panel raised the concern of confusing the patient with the possibility that ICS remains suspended even when OCS course is concluded.

#### 4.3.1. Beta Agonist

**Recommendation 3.** The first-line treatment for acute asthma attack is SABA, which can be administered three times every 20 min via a pMDI and spacer or by a nebulizer. In case of a severe episode and need for oxygen therapy, nebulization should be preferred. LABA should be stopped when SABA is used more frequently than every 4 h. Intravenous salbutamol is not recommended but can be considered in an intensive care setting in case of failed response to first-line therapy.

#### 4.3.2. Ipratropium Bromide

**Recommendation 4.** In case of moderate/severe asthma attack or in case of poor response to SABA, ipratropium bromide is recommended in combination with SABA at a dosage of 125–250 mcg/dose in patients <4 years of age and 250–500 mcg/dose in those ≥4 years of age every 20–30 min in the first hour and then every 8 h.

#### 4.3.3. Systemic Steroids

**Recommendation 5.** Early use (within 60 min after first medical examination) of systemic corticosteroids in acute asthma exacerbation can reduce the use of SABA, the risk of hospitalization and relapse of symptoms without adverse effects and regardless of the route of administration. The oral route is as effective as the IV route. The latter should be reserved for patients with vomit, too dyspneic to take oral medications or require ventilation. The effectiveness of steroids is comparable. An advantage of dexamethasone is a single or double daily dose (0.3 mg/kg once or twice daily) and a minor incidence of vomiting.

#### 4.3.4. ICS

**Recommendation 6.** In the case of mild asthma attack, it is recommended to start or continue maintenance therapy with ICS. In the case of severe asthma attack, high dose ICS in addition to systemic steroids within the first hour after arrival in ER may be associated with a reduction in the risk of hospitalization, but the evidence is insufficient to recommend this approach.

#### 4.3.5. Aminophylline and Magnesium Sulphate

**Recommendation 7.** Intravenous aminophylline and magnesium sulfate are not recommended in mild/moderate asthma attacks. In a severe acute asthma attack, when the response to first-line therapy is poor, aminophylline and magnesium sulfate can be considered in an intensive care setting.

#### 4.3.6. Leukotriene Receptor Antagonists and Inhaled Epinephrine

**Recommendation 8.** In acute asthma attacks, leukotriene receptor antagonists and inhaled epinephrine are not recommended. Inhaled epinephrine is recommended in case of bronchospasm secondary to anaphylaxis.

#### 4.3.7. Antibiotics

**Recommendation 9.** Antibiotics in acute asthma attacks are not recommended because most of these attacks are associated with a viral infection.

### 4.4. Oxygen Therapy and Types of Ventilation

The best predictive factor for hospitalization is SpO_2_ between 92% and 94% after the first hour of treatment with SABA [42,61,62,63]. There is no consensus about the SpO_2_ target to be considered to start oxygen therapy. The recent Italian guidelines on the management of asthma attacks recommended starting oxygen therapy via Venturi mask when SpO_2_ < 92% [19] while British Thoracic Society guidelines and GINA recommendations suggested starting with a mask or nasal cannula when SpO_2_ < 94% in order to achieve target saturations between 94 and 98% [3,11]. In the second round, the percentage of participants who considered starting oxygen therapy when SpO_2_ is <95% increased from 40.5 to 81%, reaching full agreement.

The choice of the interface to administer oxygen therapy depends on age, anatomical conformation of the face, patient’s compliance and severity of the respiratory distress. When a patient is unable to maintain SpO_2_ > 95% with nasal cannula or face mask, an alternative modality of oxygen delivery can be considered, including high flow oxygen (HFNC) or non-invasive ventilatory support [3,11,19], but the evidence is lacking about when to start and about which type of ventilation has to be preferred [64,65,66,67,68,69].

**Recommendation 10.** Oxygen therapy administration should be started when SpO_2_ is <95%. When standard oxygen therapy with nasal cannula or face mask is not sufficient to maintain SpO_2_ between 94 and 98%, HFNC or other methods of non-invasive ventilation can be considered.

### 4.5. Intensivist Consultation

The leading signs of severity and impending respiratory failure are the inability to speak, drowsiness, marked cyanosis not sensitive to correct therapy and persistence of tachypnea followed by bradypnea or presence of gasping [11,70,71]. In the second round, the agreement about calling for an intensivist when the child is unable to speak increased from 67.6 to 85.7%.

Children with a previous history of severe respiratory failure and hospitalization in ICU are at risk of a new episode of severe asthma attack [45,46].

Hypoxemia (pO_2_ < 60 mmHg), hypercapnia (pCO_2_ > 45 mmHg) and acidosis (pH < 7.25) at the arterial blood gas analysis are clinical signs of extreme severity [3,70,71]. In the absence of these values, the persistence of SpO_2_ < 92% despite oxygen supplementation and SABA must be considered as a clinical sign of severity and can be associated with high pCO2 values [44].

Non-invasive ventilation (NIV) offers an alternative to mechanical intubation for the management of acute respiratory failure and, theoretically, should reduce the risk of complications associated with endotracheal intubation, in particular the risk of barotrauma.

Although there is some evidence on the safety and feasibility of NIV in childhood, recent guidelines do not consider this evidence to be sufficient to make a recommendation [3,11].

Several clinical scores, such as the PRAM (Preschool Respiratory Assessment Measure) and the PASS (Pediatric Asthma Severity Score), have been proposed to evaluate the severity of asthma exacerbations in pediatric age [41], but their use is probably preferred in controlled studies rather than in clinical practice. Table 5 reports the criteria for which the patient should be admitted/transferred to the ICU.

**Recommendation 11.** Patients with severe asthma showing progressive clinical worsening despite first-line therapy signs, symptoms of respiratory failure and history of previous admissions to ICU for asthma, need an evaluation by an intensive care physician. Hypoxemia (pO_2_ < 60 mmHg), hypercapnia (pCO_2_ > 45 mmHg) and acidosis (pH < 7.25) at the arterial blood gas analysis and FiO_2_ > 50% are clinical signs of extreme severity.

### 4.6. Chest X-ray and Chest Ultrasound

Chest X-ray does not modify clinical approach and therapeutic decision in children with acute asthma attack [72,73,74]. In a few cases of patients with SpO_2_ ≤ 92% or fever, chest X-rays showed pneumonia, atelectasis or pneumothorax [74]. Chest X-ray may also help in differential diagnosis, such as respiratory infections, foreign body inhalation or congenital diseases such as heart disease, vascular ring or congenital lobar emphysema [75,76,77].

To date, there is no evidence to recommend in children a lung ultrasound during an acute asthma attack. During an exacerbation, chest ultrasound can give false-positive results for pneumothorax [78]; in the second round, the number of participants who did not consider appropriate a lung ultrasound in the suspect of this condition increased from 58 to 76%.

**Recommendation 12.** During asthma attack, chest X-rays and chest ultrasound are not recommended. When pneumothorax, atelectasis or pneumonia are suspected or when the child is poorly responsive to treatment, chest X-rays can be considered.

### 4.7. Arterial Blood Gas Analysis and Blood Tests

A prospective study showed that patients with SpO_2_ ≥ 92% did not develop respiratory failure, and therefore, arterial blood gas analysis is not indicated [44]. On the other hand, values of SpO_2_ < 92% are associated with severe attacks and the risk of hypercapnia [11,43,79,80]. A pCO_2_ value >42 mmHg is associated with a severe asthma attack [19]. In the second round, a significant increase in the agreement about considering arterial blood gas analysis not appropriate in all asthma attacks has been reported (41.9% vs. 81%).

Blood tests are usually not recommended. When high doses of inhaled or intravenous salbutamol are administered, blood potassium levels should be monitored for the risk of hypokalemia [11,19,81,82].

**Recommendation 13.** Arterial blood gas analysis and blood tests are not routinely recommended. Arterial blood gas analysis can be considered when the attack is severe, the child is poorly responsive to treatment and SpO_2_ is <92%. Blood tests can be considered when high doses of SABA are administered to check for hypokalemia.

### 4.8. Lung Function Test

Spirometry is the gold standard test both for diagnosis and follow-up of asthma disease and after the second round the percentage of participants who considered spirometry an important tool to diagnose asthma increased from 46 to 74.6%, almost reaching full consensus. During an acute asthma attack, the measurement of lung function by FEV_1_ (forced expiratory volume in 1 s) or PEF is strongly recommended whenever possible, without delaying therapy. These tests define the severity of the attack (mild for PEF or FEV_1_ values >80%, moderate for values between 60 and 80% and severe if PEF or FEV_1_ are <60%) and assess the response to therapy [19,20,21,22,23,24,25,26,27,28,29,30,31,32,33,34,35,36,37,38,39,40,41,42,43,44,45,46,47,48,49,50,51,52,53,54,55,56,57,58,59,60,61,62,63,64,65,66,67,68,69,70,71,72,73,74,75,76,77,78,79,80,81,82,83].

Although PEF is mentioned in several guidelines and recommendations to diagnose asthma, its use in clinical practice is less common than spirometry. In addition, PEF is indicated for children who are already familiar with the technique and the result should be expressed as a percentage of the patient’s best PEF value (personal best) to increase its reliability [84].

The following criteria were proposed to discriminate between patients who need hospitalization and those who can be discharged at home [85]:

- In case of FEV_1_ < 40% predicted or PEF < 40% of the personal best after one hour of treatment, hospitalization is indicated.
- For FEV_1_ or PEF values between 40–60% after one hour of treatment, discharge can be considered after having taken account of risk factors for asthma-related death and availability of adequate follow-up.
- For FEV_1_ or PEF values >60%, discharge is likely, always after evaluation of any associated risk factors and only when an adequate follow-up is planned.
- Given the importance of spirometry, pediatricians and nurses from both hospital and primary care should be trained to perform this test [11,86,87].

**Recommendation 14.** During asthma attack, pulmonary function tests are recommended to define the severity of the attack and assess response to treatment.

### 4.9. Admission to the Hospital

Hospitalization in case of acute asthma attack is not always necessary and the percentage of participants in agreement with this increased from 52.7 to 76.2% in the second round. To avoid inappropriate hospitalizations, an adequate risk stratification is fundamental to identify which patients need hospital admission and which ones can be managed at home.

The need for oxygen therapy is one of the main indications for hospitalization and values of SpO_2_ < 92% in room air at the time of the first clinical examination are good predictors of hospitalization [42]. Furthermore, patients with poor response to first-line therapy, clinical deterioration in the first hour of treatment, pneumothorax, atelectasis or pneumonia need hospitalization.

**Recommendation 15.** Admission to the hospital is recommended when the response to treatment is poor, especially within the first hour and in case of pneumothorax, atelectasis or pneumonia. Oxygen therapy is the first indication for admission. Pulmonary function tests can be useful to define the severity of the attack. History of severe attacks, a home far from the hospital and poor compliance are factors to be considered for the need for hospitalization.

### 4.10. Follow-Up after an Asthma Attack

Specialist respiratory follow-up in asthmatic patients is associated with a reduction in hospitalizations and emergency department visits, with better quality of life, better symptom control, increased asthma knowledge, less need for SABA and higher patient satisfaction [88,89,90,91]. Following an asthma attack, each patient should be evaluated by the primary care physician within 2–4 weeks or earlier (2–7 days) in case of hospitalization [3,11,19]. After an asthma attack is managed in the emergency department, the patient should be referred to the specialist within 4–6 weeks since respiratory follow-up has been associated with a reduced risk of new admissions within 90 days of the event [92]. Specialists should follow the patient who had an acute asthma attack requiring hospitalization for at least one year and long-term if the attack was severe and life-threatening [11].

Specialist respiratory follow-up aims to confirm the diagnosis of asthma, optimize therapy, assess respiratory function with spirometry and other tests where available, identify asthma phenotype (skin prick test, FeNO, blood tests) and comorbidities (sinusitis, nasal polyposis, allergic bronchopulmonary aspergillosis, severe rhinitis, vocal cord dysfunction, gastroesophageal reflux, obesity, obstructive sleep apnea syndrome), detect risk factors for asthma-related death (previous near-fatal asthmatic attacks, anaphylaxis or food allergy; psychiatric or psychosocial disorders, poor compliance), educate the patient and the caregivers to asthma management in order to improve control and reduce exacerbations [93,94,95].

In the second round, the percentage of participants who considered it appropriate to start a respiratory follow-up increased from 39 to 59% without, however, reaching full agreement.

**Recommendation 16.** After an asthma attack, the patient should be evaluated within 2–4 weeks or if the attack was managed in the emergency department or required hospitalization, within 1 week by the primary care physician and referred to the specialist within 4–6 weeks. The specialist should confirm the diagnosis of asthma, assess comorbidities and risk factors, evaluate lung function by spirometry, assess allergies, start maintenance therapy and educate patients and caregivers on asthma management (also providing an action plan).

After an asthma attack, spirometry should be performed to measure lung function, assess response to SABA and record the patient’s personal best (i.e., highest FEV_1_ value) [3]. The indications to perform spirometry to confirm asthma diagnosis and to follow-up with the patient were endorsed by a higher number of experts after the second round of the survey (from 45.9 to 74.6% and from 62.2 to 81%, respectively).

Clinical examination and spirometry can be repeated in the following weeks until the patient achieves good symptom control and normal FEV_1_ values. Indeed, FEV_1_ reaches a plateau after about 2 months of treatment with inhaled corticosteroids. Measurement of lung function is indicated 3–6 months after initiation of treatment (at this time, the patient’s personal best should be identified) [3]. Follow-up spirometry within the first 6 months of maintenance therapy has been associated with a reduced risk of asthma-related [96]. In high-risk patients and in severe asthma, spirometry can be performed more frequently [97].

**Recommendation 17.** Measurement of lung function by spirometry is indicated at the time of initial assessment, 3–6 months after the start of treatment, during exacerbations and at least every 1–2 years to verify the maintenance of good lung function.

Following an acute asthma attack, allergy assessment is crucial to support the diagnosis of asthma (>85% of asthma in children is allergic), identify the triggers that may have contributed to exacerbation and develop strategies to reduce exposure [98,99]. Skin prick test for common environmental allergens is quick, easy to perform and, when performed by experienced personnel using standardized allergen extracts, shows high sensitivity. Positive prick test, blood eosinophilia ≥ 300/μL or increased total and specific IgE can confirm atopy [11,100]; however, blood tests can be preferred over skin prick tests for non-collaborative patients, those with extensive skin disease or those with risk of anaphylaxis. There is no age limit for performing skin prick tests, but it is well known that results may change over time as the child grows up. Positive results have been reached in the second round since the percentage of participants considering skin prick test after an asthma attack increased from 64.9 to 82.5%.

FeNO is a biomarker of Th2 airway inflammation [101]. High levels of FeNO are reported in children with eosinophilic asthma [102], especially during exacerbations. Values are significantly reduced after systemic steroid therapy [103] and can also be considered when assessing adherence to inhaled steroid treatment [104,105]. To date, the role of FeNO in predicting children’s lung function is unclear, but elevated values were associated with reduced respiratory function [106] and increased risk of exacerbations [107].

**Recommendation 18.** A skin prick test for inhaled allergens is recommended in children with asthma attack to support the diagnosis, provide information on phenotype and identify triggers that could be avoided. Allergen-specific IgE blood test is a second-line test that can be considered in some cases. There is no age limit for performing prick tests. When available, FeNO should be measured at least once to identify eosinophilic inflammation (values of 35 ppb or more are considered positive), although low values do not exclude a diagnosis of asthma.

## 5. Conclusions

For a heterogeneous disease such as asthma, the application of methods aiming to increase the homogeneity of behaviors by pediatricians both in the hospital and in primary care appears useful and appropriate. Through the RAND method, the participants discussed the statements derived from the guidelines and there was a clear increase in the agreement in the management of acute asthma attack; however, agreement was not achieved in all the scenarios. It should be noted that the participants in the project came from two different clinical contexts, a condition that could have affected the responses to the different scenarios. For this reason, the results achieved demonstrate the usefulness of the RAND method for the selection of good practices and constitute the basis of an evidence-based approach. The findings obtained can establish the basis for educational interventions that aim to optimize the care of the child with asthma attack both in primary care and in the hospital. Patient-centered quality control programs developed on evidence-based medicine would ensure high-quality care, optimize the effectiveness of treatment and ultimately good asthma control in patients.

An important link between the primary care pediatrician and the pediatrician in the hospital may be the possibility of sharing spirometry results, also including the use of new technologies such as telemedicine. The treatment of the child with an asthma attack is not concluded with the treatment of the acute attack but must continue with the clinical and instrumental follow-up. The spirometer in the pediatrician’s office could also help in the diagnosis of mild symptoms of asthma. On the other hand, a cornerstone of the treatment of pediatric asthma is the prevention of exacerbations. Monitoring the progress of asthma through spirometry could allow the pediatrician in the primary care to intervene early by modifying the maintenance therapy and help the patient to achieve good control of the disease. Moreover, considering the results of studies that showed the effect of particulate matter in inducing asthma exacerbation events leading to emergency room admission or hospitalization [108,109], further research should analyze the impact of air pollutants in the management of asthma attacks in pediatric age.

## Figures and Tables

**Table 1:** Classification of median and disagreement of experts on different scenarios.

Median	Disagreement	Classification
7–9	No	Appropriate with agreement
7–9	Yes	Appropriate but with disagreement
4–6	Not applicable	Uncertain
1–3	Not applicable	Not appropriate

The mean and disagreement were classified into three levels of appropriateness (appropriate: between ‘7’ and ‘9’, without disagreement; uncertain: between ‘4’ and ‘6’ or any median with disagreement; inappropriate: between ‘1’ and ‘3’ with agreement).

**Table 2:** Severity of asthma attack based on clinical criteria, lung function, SpO_2_ and arterial blood analysis.

**MILD**	**MODERATE**	**SEVERE**
- SpO_2_ >95% in room air - normal HR - PEF-FEV_1_ >80% predicted - pCO_2_ <38 mmHg - normal consciousness - normal skin color - able to speak - normal RR - no use of accessory respiratory muscles - teleinspiratory wheezing at chest auscultation	- SpO_2_ 92–95% in room air - increased HR - PEF-FEV_1_ 60–80% predicted - pCO_2_ 38–42 mmHg - agitation - pale appearance - able to speak - increased RR - moderate use of accessory respiratory muscles - espiratory wheezing at chest auscultation	- SpO_2_ <92% in room air - increased HR - PEF-FEV_1_ <60% predicted - pCO_2_ >42 mmHg - altered consciousness - pale appearance or cyanosis - difficulty speaking - increased RR - severe use of accessory respiratory muscles - in and espiratory wheezing at chest auscultation or silent chest
**Normal range for age**		
RR <2 months <60/min 2–12 months <50/min >1–5 years <40/min 6–9 years <30/min 10–14 years <20/min	HR 0–12 months <160 bpm 1–2 years <120 bpm 2–8 years <100 bpm	

Reprinted from ref. [20]. One parameter is sufficient to classify the patient in one of the three classes. SpO_2_, oxygen saturation; HR, heart rate; PEF, peak expiratory flow; FEV_1_, forced expiratory volume in 1 s; RR respiratory rate; pCO_2_ partial pressure of carbon dioxide.

**Table 3:** Medical history in a child with asthma.

Medical History Should Include:
Onset and trigger (if known) of exacerbation
Severity of asthma symptoms including physical activity limitation or sleep disturbances
Signs of anaphylaxis
Risk factors for asthma-death
Risk factors related to persistent airflow limitation such as preterm birth, low birth weight, pulmonary bronchodysplasia, associated diseases and chronic mucus hypersecretion
Treatments including doses and devices, pattern of adherence, any recent dose changes and response to current therapy

**Table 4:** Risk factors for severe asthma attack and respiratory failure.

Risk Factors for Severe Asthma Attack with Respiratory Failure and Death
History of severe asthma attack with respiratory failure and need of invasive or non-invasive ventilation [40,41]
Access to ER or hospitalization for asthma over the past 12 months [41]
Recent OCS course [40]
No maintenance therapy with ICS [42]
SABA overuse (more than 1 canister/month) [43,44]
Poor adherence to ICS treatment and no written action plan [40,45,46]
Food allergy [47,48]
Smoking exposure or exposure to allergens or pollution [40,49]
Psychiatric disorders and/or social issues, poor family compliance [50]
Comorbidities such as obesity [51], pneumonia, diabetes and cardiac arrhythmias [52]

ER, emergency room; OCS, oral corticosteroids; ICS, inhaled corticosteroids; SABA, short-acting beta2 agonist.

**Table 5:** Criteria for hospitalization/transfer to the ICU for a patient with acute asthma attack.

Criteria for Hospitalization and Transfer to the ICU for a Patient with Acute Asthma Attack
Need for ventilatory support
Severe asthma attack unresponsive to therapy [5]: Worsening of PEF or FEV_1_ (PEF and FEV_1_ <40% predicted or patient’s personal best). Persistent or worsening hypoxia (SpO_2_ < 90–92%, pO_2_ < 60 mmHg). Hypercapnia (pCO_2_ > 45 mmHg). Acidosis (pH < 7.25). High probability of respiratory failure (muscle exhaustion, persistent tachycardia, persistent tachypnoea that evolves into bradypnea, gasping, absent air entry to auscultation). Altered consciousness. Presence of complications (pneumothorax, atelectasis, pneumonia).

PEF, peak expiratory flow; FEV_1_, forced expiratory volume in 1 s; SpO_2_, oxygen saturation; pO_2_ partial pressure of oxygen; pCO_2_ partial pressure of carbon dioxide.

## Data Availability

The data presented in this study are available in this article.

## Supplementary Materials

The following are available online at https://www.mdpi.com/article/10.3390/ijerph182312775/s1, Supplementary Material S1: Questionnaire on the management of acute asthma attack in children, Supplementary Material S2: Graphs PRE POST.
