# Supplementary material for: Management of Children with Acute Asthma Attack: A RAND/UCLA Appropriateness Approach
Source: Int J Environ Res Public Health. 2021 Dec 3;18(23):12775. doi: 10.3390/ijerph182312775 (PMC8657661; doi:10.3390/ijerph182312775)
Supplement: Supplementary file 1 [file ijerph-18-12775-s001.zip › Suppl_1_Questionnaire.pdf]

Questionnaire on the management of acute asthma attack in children

For each scenario (indicated by numbers 1 to 13) there may be more than one appropriate or inappropriate answer (indicated by letters)

1) In case of acute asthma attack, the patient already known for asthma

a. should be invited to take inhaled therapy with short-acting beta-2 agonist (SABA)
- never appropriate
- hardly ever appropriate
- usually not appropriate
- sometimes appropriate
- often appropriate
- very often appropriate
- usually appropriate
- almost always appropriate
- always appropriate

b. Inhaled steroids should be continued for patients who already use inhaled steroids as maintenance therapy
- never appropriate
- hardly ever appropriate
- usually not appropriate
- sometimes appropriate
- often appropriate
- very often appropriate
- usually appropriate
- almost always appropriate
- always appropriate

c. should be invited to take oral steroids first
- never appropriate
- hardly ever appropriate
- usually not appropriate
- sometimes appropriate
- often appropriate
- very often appropriate
- usually appropriate
- almost always appropriate
- always appropriate

d. must be immediately invited to go to the hospital/paediatric emergency department
- never appropriate
- hardly ever appropriate
- usually not appropriate
- sometimes appropriate
- often appropriate
- very often appropriate
- usually appropriate
- almost always appropriate
- always appropriate

2) In case of clinical finding of acute asthma attack, the patient must be sent to the hospital/paediatric emergency department:

a. if SpO_2 <95%
- never appropriate
- hardly ever appropriate
- usually not appropriate
- sometimes appropriate
- often appropriate
- very often appropriate
- usually appropriate
- almost always appropriate
- always appropriate

b. if SpO_2 <95%, inhalation therapy with SABA must be administered before going to the paediatric emergency department
- never appropriate
- hardly ever appropriate
- usually not appropriate
- sometimes appropriate
- often appropriate
- very often appropriate
- usually appropriate
- almost always appropriate
- always appropriate

c. if SpO_2 <92%
- never appropriate
- hardly ever appropriate
- usually not appropriate
- sometimes appropriate
- often appropriate
- very often appropriate
- usually appropriate
- almost always appropriate
- always appropriate

d. if the patient has difficulty speaking
- never appropriate
- hardly ever appropriate
- usually not appropriate
- sometimes appropriate
- often appropriate
- very often appropriate
- usually appropriate
- almost always appropriate
- always appropriate

e. if there is alteration of the state of consciousness
- never appropriate
- hardly ever appropriate
- usually not appropriate

- sometimes appropriate
- often appropriate
- very often appropriate
- usually appropriate
- almost always appropriate
- always appropriate

f. if the patient has a history of previous severe asthma attacks that required hospitalization
- never appropriate
- hardly ever appropriate
- usually not appropriate
- sometimes appropriate
- often appropriate
- very often appropriate
- usually appropriate
- almost always appropriate
- always appropriate

g. if there is poor response after inhalation therapy with SABA
- never appropriate
- hardly ever appropriate
- usually not appropriate
- sometimes appropriate
- often appropriate
- very often appropriate
- usually appropriate
- almost always appropriate
- always appropriate

h. if the patient has known allergies
- never appropriate
- hardly ever appropriate
- usually not appropriate
- sometimes appropriate
- often appropriate
- very often appropriate
- usually appropriate
- almost always appropriate
- always appropriate

i. if the patient has underlying diseases and/or pulmonary or extrapulmonary risk factors (e.g. prematurity, pulmonary bronchodysplasia, bronchiectasis, congenital malformations involving airways, heart disease, obesity, psychiatric disorders)
- never appropriate
- hardly ever appropriate
- usually not appropriate
- sometimes appropriate
- often appropriate
- very often appropriate
- usually appropriate
- almost always appropriate
- always appropriate

j. if poor family compliance is suspected
● never appropriate
● hardly ever appropriate
● usually not appropriate
● sometimes appropriate
● often appropriate
● very often appropriate
● usually appropriate
● almost always appropriate
● always appropriate

3) What is the therapy in case of an acute asthma attack?

a. SABA is the drug of choice
● never appropriate
● hardly ever appropriate
● usually not appropriate
● sometimes appropriate
● often appropriate
● very often appropriate
● usually appropriate
● almost always appropriate
● always appropriate

b. SABA should be administered 3 times every 20 minutes
● never appropriate
● hardly ever appropriate
● usually not appropriate
● sometimes appropriate
● often appropriate
● very often appropriate
● usually appropriate
● almost always appropriate
● always appropriate

c. therapy with long-acting beta-2 agonists (LABA) should be suspended when SABA is taken for more than every 4 hours
● never appropriate
● hardly ever appropriate
● usually not appropriate
● sometimes appropriate
● often appropriate
● very often appropriate
● usually appropriate
● almost always appropriate
● always appropriate

d. therapy with SABA can be administered either by nebulizer or metered dose inhaler (MDI) with spacer
● never appropriate
● hardly ever appropriate

- usually not appropriate
- sometimes appropriate
- often appropriate
- very often appropriate
- usually appropriate
- almost always appropriate
- always appropriate

e. SABA should be taken via nebulizer only in case of poor improvement after therapy with MDI and spacer
- never appropriate
- hardly ever appropriate
- usually not appropriate
- sometimes appropriate
- often appropriate
- very often appropriate
- usually appropriate
- almost always appropriate
- always appropriate

f. SABA should be taken by nebulizer in case of oxygen supplementation
- never appropriate
- hardly ever appropriate
- usually not appropriate
- sometimes appropriate
- often appropriate
- very often appropriate
- usually appropriate
- almost always appropriate
- always appropriate

g. antibiotics must always be started
- never appropriate
- hardly ever appropriate
- usually not appropriate
- sometimes appropriate
- often appropriate
- very often appropriate
- usually appropriate
- almost always appropriate
- always appropriate

h. systemic steroid therapy should always be started
- never appropriate
- hardly ever appropriate
- usually not appropriate
- sometimes appropriate
- often appropriate
- very often appropriate
- usually appropriate
- almost always appropriate
- always appropriate

i. systemic steroid therapy should always be administered orally
- never appropriate
- hardly ever appropriate
- usually not appropriate
- sometimes appropriate
- often appropriate
- very often appropriate
- usually appropriate
- almost always appropriate
- always appropriate

j. systemic steroid therapy should always be administered intravenously
- never appropriate
- hardly ever appropriate
- usually not appropriate
- sometimes appropriate
- often appropriate
- very often appropriate
- usually appropriate
- almost always appropriate
- always appropriate

k. steroid therapy should be administered intravenously only in most severe cases
- never appropriate
- hardly ever appropriate
- usually not appropriate
- sometimes appropriate
- often appropriate
- very often appropriate
- usually appropriate
- almost always appropriate
- always appropriate

l. steroid therapy should be administered intravenously in case of impossibility to oral administration (e.g. vomits, altered state of consciousness)
- never appropriate
- hardly ever appropriate
- usually not appropriate
- sometimes appropriate
- often appropriate
- very often appropriate
- usually appropriate
- almost always appropriate
- always appropriate

m. the effects of systemic steroid therapy are observed over 3-4 hours
- never appropriate
- hardly ever appropriate
- usually not appropriate
- sometimes appropriate
- often appropriate

- very often appropriate
- usually appropriate
- almost always appropriate
- always appropriate

n. the first choice steroid therapy is betamethasone
- never appropriate
- hardly ever appropriate
- usually not appropriate
- sometimes appropriate
- often appropriate
- very often appropriate
- usually appropriate
- almost always appropriate
- always appropriate

o. the first choice steroid therapy is prednisone
- never appropriate
- hardly ever appropriate
- usually not appropriate
- sometimes appropriate
- often appropriate
- very often appropriate
- usually appropriate
- almost always appropriate
- always appropriate

p. the first choice steroid therapy is desamethasone
- never appropriate
- hardly ever appropriate
- usually not appropriate
- sometimes appropriate
- often appropriate
- very often appropriate
- usually appropriate
- almost always appropriate
- always appropriate

q. there is no evidence that intravenous administration of steroids has more advantages than oral administration
- never appropriate
- hardly ever appropriate
- usually not appropriate
- sometimes appropriate
- often appropriate
- very often appropriate
- usually appropriate
- almost always appropriate
- always appropriate

r. steroid therapy should be continued for at least 2-3 days
- never appropriate

- hardly ever appropriate
- usually not appropriate
- sometimes appropriate
- often appropriate
- very often appropriate
- usually appropriate
- almost always appropriate
- always appropriate

s. steroid therapy should be continued for at least 3-5 days
- never appropriate
- hardly ever appropriate
- usually not appropriate
- sometimes appropriate
- often appropriate
- very often appropriate
- usually appropriate
- almost always appropriate
- always appropriate

t. there is no need for tapering off if the steroid is used for a week or less
- never appropriate
- hardly ever appropriate
- usually not appropriate
- sometimes appropriate
- often appropriate
- very often appropriate
- usually appropriate
- almost always appropriate
- always appropriate

u. steroid therapy must always be tapered off gradually
- never appropriate
- hardly ever appropriate
- usually not appropriate
- sometimes appropriate
- often appropriate
- very often appropriate
- usually appropriate
- almost always appropriate
- always appropriate

v. therapy with anticholinergics (ipratropium bromide) should always be administered
- never appropriate
- hardly ever appropriate
- usually not appropriate
- sometimes appropriate
- often appropriate
- very often appropriate
- usually appropriate
- almost always appropriate

● always appropriate

w. therapy with anticholinergics (ipratropium bromide) should be administered in case of poor response to SABA
● never appropriate
● hardly ever appropriate
● usually not appropriate
● sometimes appropriate
● often appropriate
● very often appropriate
● usually appropriate
● almost always appropriate
● always appropriate

x. therapy with anticholinergics (ipratropium bromide) should be administered 3 times every 20 minutes
● never appropriate
● hardly ever appropriate
● usually not appropriate
● sometimes appropriate
● often appropriate
● very often appropriate
● usually appropriate
● almost always appropriate
● always appropriate

y. inhaled epinephrine should be administered in the event of a severe asthmatic attack
● never appropriate
● hardly ever appropriate
● usually not appropriate
● sometimes appropriate
● often appropriate
● very often appropriate
● usually appropriate
● almost always appropriate
● always appropriate

z. inhaled epinephrine should be administered in case of poor response to first line therapy (SABA, systemic steroid, ipratropium bromide)
● never appropriate
● hardly ever appropriate
● usually not appropriate
● sometimes appropriate
● often appropriate
● very often appropriate
● usually appropriate
● almost always appropriate
● always appropriate

za. in case of poor response to first-line therapy (SABA, systemic steroid, ipratropium bromide) intravenous aminophylline should be administered
● never appropriate

- hardly ever appropriate
- usually not appropriate
- sometimes appropriate
- often appropriate
- very often appropriate
- usually appropriate
- almost always appropriate
- always appropriate

zb. in case of poor response to first-line therapy (SABA, systemic steroid, ipratropium bromide) intravenous magnesium sulphate should be administered

- never appropriate
- hardly ever appropriate
- usually not appropriate
- sometimes appropriate
- often appropriate
- very often appropriate
- usually appropriate
- almost always appropriate
- always appropriate

zc. in case of poor response to first-line therapy (SABA, systemic steroid, ipratropium bromide) leukotriene receptor antagonist should be administered

- never appropriate
- hardly ever appropriate
- usually not appropriate
- sometimes appropriate
- often appropriate
- very often appropriate
- usually appropriate
- almost always appropriate
- always appropriate

zd. In case of an acute asthma attack it's always needed inhaled steroid therapy

- never appropriate
- hardly ever appropriate
- usually not appropriate
- sometimes appropriate
- often appropriate
- very often appropriate
- usually appropriate
- almost always appropriate
- always appropriate

ze. in case of a moderate/severe acute asthma attack, oral steroid therapy replaces inhaled steroid therapy, that should be stopped

- never appropriate
- hardly ever appropriate
- usually not appropriate
- sometimes appropriate
- often appropriate

- very often appropriate
- usually appropriate
- almost always appropriate
- always appropriate

4) Oxygen therapy in case of acute asthma attack

a. is always indicated
- never appropriate
- hardly ever appropriate
- usually not appropriate
- sometimes appropriate
- often appropriate
- very often appropriate
- usually appropriate
- almost always appropriate
- always appropriate

b. is indicated if SpO_2 <95%
- never appropriate
- hardly ever appropriate
- usually not appropriate
- sometimes appropriate
- often appropriate
- very often appropriate
- usually appropriate
- almost always appropriate
- always appropriate

c. is indicated if SpO_2 <92%
- never appropriate
- hardly ever appropriate
- usually not appropriate
- sometimes appropriate
- often appropriate
- very often appropriate
- usually appropriate
- almost always appropriate
- always appropriate

d. must be applied through nasal cannulae
- never appropriate
- hardly ever appropriate
- usually not appropriate
- sometimes appropriate
- often appropriate
- very often appropriate
- usually appropriate
- almost always appropriate
- always appropriate

e. must be applied using a Venturi-mask

- never appropriate
- hardly ever appropriate
- usually not appropriate
- sometimes appropriate
- often appropriate
- very often appropriate
- usually appropriate
- almost always appropriate
- always appropriate

f. must be applied using a mask with reservoir
- never appropriate
- hardly ever appropriate
- usually not appropriate
- sometimes appropriate
- often appropriate
- very often appropriate
- usually appropriate
- almost always appropriate
- always appropriate

5) In case of an acute asthma attack with poor response to standard oxygen therapy

a. High Flow Oxygen Therapy (HFNC) should be started
- never appropriate
- hardly ever appropriate
- usually not appropriate
- sometimes appropriate
- often appropriate
- very often appropriate
- usually appropriate
- almost always appropriate
- always appropriate

b. non-invasive ventilation with continuous positive airway pressure (CPAP) should be initiated
- never appropriate
- hardly ever appropriate
- usually not appropriate
- sometimes appropriate
- often appropriate
- very often appropriate
- usually appropriate
- almost always appropriate
- always appropriate

6) The patient needs intensive care evaluation for possible transfer to intensive care unit (ICU) in case of:

a. child's agitation/inability to speak
- never appropriate
- hardly ever appropriate

- usually not appropriate
- sometimes appropriate
- often appropriate
- very often appropriate
- usually appropriate
- almost always appropriate
- always appropriate

b. sleepy state
- never appropriate
- hardly ever appropriate
- usually not appropriate
- sometimes appropriate
- often appropriate
- very often appropriate
- usually appropriate
- almost always appropriate
- always appropriate

c. history of previous hospitalization in PICU for asthma and/or respiratory failure
- never appropriate
- hardly ever appropriate
- usually not appropriate
- sometimes appropriate
- often appropriate
- very often appropriate
- usually appropriate
- almost always appropriate
- always appropriate

d. marked cyanosis not responsive to therapy
- never appropriate
- hardly ever appropriate
- usually not appropriate
- sometimes appropriate
- often appropriate
- very often appropriate
- usually appropriate
- almost always appropriate
- always appropriate

e. persistent tachypnea (>3 hours) followed by bradypnea
- never appropriate
- hardly ever appropriate
- usually not appropriate
- sometimes appropriate
- often appropriate
- very often appropriate
- usually appropriate
- almost always appropriate

- always appropriate

f. absence of chest sounds (silent chest) associated with tachypnea/dyspnoea
- never appropriate
- hardly ever appropriate
- usually not appropriate
- sometimes appropriate
- often appropriate
- very often appropriate
- usually appropriate
- almost always appropriate
- always appropriate

g. presence of agonic breathing (gasping)
- never appropriate
- hardly ever appropriate
- usually not appropriate
- sometimes appropriate
- often appropriate
- very often appropriate
- usually appropriate
- almost always appropriate
- always appropriate

h. Pediatric Asthma Severity Score (PASS) >6
- never appropriate
- hardly ever appropriate
- usually not appropriate
- sometimes appropriate
- often appropriate
- very often appropriate
- usually appropriate
- almost always appropriate
- always appropriate

i. Persistent SpO_2 <92% despite oxygen therapy with reservoir lasting >3 hours and despite SABA
- never appropriate
- hardly ever appropriate
- usually not appropriate
- sometimes appropriate
- often appropriate
- very often appropriate
- usually appropriate
- almost always appropriate
- always appropriate

j. pO_2 <60 mmHg at arterial blood gas analysis
- never appropriate
- hardly ever appropriate
- usually not appropriate
- sometimes appropriate
- often appropriate

● very often appropriate
● usually appropriate
● almost always appropriate
● always appropriate

k. pCO_2 >45 mmHg at arterial blood gas analysis
● never appropriate
● hardly ever appropriate
● usually not appropriate
● sometimes appropriate
● often appropriate
● very often appropriate
● usually appropriate
● almost always appropriate
● always appropriate

l. FiO_2 >50%
● never appropriate
● hardly ever appropriate
● usually not appropriate
● sometimes appropriate
● often appropriate
● very often appropriate
● usually appropriate
● almost always appropriate
● always appropriate

m. need for positive pressure respiratory support (both invasive and non-invasive)
● never appropriate
● hardly ever appropriate
● usually not appropriate
● sometimes appropriate
● often appropriate
● very often appropriate
● usually appropriate
● almost always appropriate
● always appropriate

n. barotrauma or lung disease evident at chest X-rays or chest ultrasound
● never appropriate
● hardly ever appropriate
● usually not appropriate
● sometimes appropriate
● often appropriate
● very often appropriate
● usually appropriate
● almost always appropriate
● always appropriate

7) In case of acute asthma attack, chest X-rays

a. must always be performed

- never appropriate
- hardly ever appropriate
- usually not appropriate
- sometimes appropriate
- often appropriate
- very often appropriate
- usually appropriate
- almost always appropriate
- always appropriate

b. must be performed in case of failure to respond to SABA
- never appropriate
- hardly ever appropriate
- usually not appropriate
- sometimes appropriate
- often appropriate
- very often appropriate
- usually appropriate
- almost always appropriate
- always appropriate

c. should always be performed if the patient is febrile
- never appropriate
- hardly ever appropriate
- usually not appropriate
- sometimes appropriate
- often appropriate
- very often appropriate
- usually appropriate
- almost always appropriate
- always appropriate

d. should always be performed if the patient requires oxygen therapy
- never appropriate
- hardly ever appropriate
- usually not appropriate
- sometimes appropriate
- often appropriate
- very often appropriate
- usually appropriate
- almost always appropriate
- always appropriate

e. must always be performed in case of hospitalization
- never appropriate
- hardly ever appropriate
- usually not appropriate
- sometimes appropriate
- often appropriate
- very often appropriate
- usually appropriate
- almost always appropriate

● always appropriate

f. should be performed if the patient has underlying diseases and pulmonary or extrapulmonary risk factors (e.g. prematurity, pulmonary bronchodysplasia, bronchiectasis, congenital malformations involving the airways, heart disease)
● never appropriate
● hardly ever appropriate
● usually not appropriate
● sometimes appropriate
● often appropriate
● very often appropriate
● usually appropriate
● almost always appropriate
● always appropriate

8) In case of acute asthma, chest ultrasound

a. must be performed if pneumothorax is suspected
● never appropriate
● hardly ever appropriate
● usually not appropriate
● sometimes appropriate
● often appropriate
● very often appropriate
● usually appropriate
● almost always appropriate
● always appropriate

b. must be performed if pneumonia is suspected
● never appropriate
● hardly ever appropriate
● usually not appropriate
● sometimes appropriate
● often appropriate
● very often appropriate
● usually appropriate
● almost always appropriate
● always appropriate

9) In the event of an acute asthma attack, arterial blood gas analysis

a. is always indicated
● never appropriate
● hardly ever appropriate
● usually not appropriate
● sometimes appropriate
● often appropriate
● very often appropriate
● usually appropriate
● almost always appropriate
● always appropriate

b. is indicated in patients with pre-existing lung diseases (pulmonary bronchodysplasia, bronchiectasis, heart disease)
● never appropriate
● hardly ever appropriate
● usually not appropriate
● sometimes appropriate
● often appropriate
● very often appropriate
● usually appropriate
● almost always appropriate
● always appropriate

c. is indicated if the patient requires oxygen therapy
● never appropriate
● hardly ever appropriate
● usually not appropriate
● sometimes appropriate
● often appropriate
● very often appropriate
● usually appropriate
● almost always appropriate
● always appropriate

10) In the event of an acute asthma attack, blood tests should be performed

a. always indicated
● never appropriate
● hardly ever appropriate
● usually not appropriate
● sometimes appropriate
● often appropriate
● very often appropriate
● usually appropriate
● almost always appropriate
● always appropriate

b. indicated in case of hospitalization of the patient
● never appropriate
● hardly ever appropriate
● usually not appropriate
● sometimes appropriate
● often appropriate
● very often appropriate
● usually appropriate
● almost always appropriate
● always appropriate

c. indicated if the patient is febrile
● never appropriate
● hardly ever appropriate
● usually not appropriate
● sometimes appropriate

- often appropriate
- very often appropriate
- usually appropriate
- almost always appropriate
- always appropriate

11) In case of acute asthmatic attack

a. spirometry is always useful for diagnosis
- never appropriate
- hardly ever appropriate
- usually not appropriate
- sometimes appropriate
- often appropriate
- very often appropriate
- usually appropriate
- almost always appropriate
- always appropriate

b. spirometry is useful in patients already diagnosed with asthma
- never appropriate
- hardly ever appropriate
- usually not appropriate
- sometimes appropriate
- often appropriate
- very often appropriate
- usually appropriate
- almost always appropriate
- always appropriate

c. the use of peak expiratory flow (PEF) is always useful for diagnosis
- never appropriate
- hardly ever appropriate
- usually not appropriate
- sometimes appropriate
- often appropriate
- very often appropriate
- usually appropriate
- almost always appropriate
- always appropriate

d. the use of PEF useful in patients already diagnosed with asthma
- never appropriate
- hardly ever appropriate
- usually not appropriate
- sometimes appropriate
- often appropriate
- very often appropriate
- usually appropriate
- almost always appropriate

● always appropriate

e. the use of PEF in patients already followed for asthma is useful to define the severity of asthma
● never appropriate
● hardly ever appropriate
● usually not appropriate
● sometimes appropriate
● often appropriate
● very often appropriate
● usually appropriate
● almost always appropriate
● always appropriate

f. PEF <50% of personal best indicates a severe attack
● never appropriate
● hardly ever appropriate
● usually not appropriate
● sometimes appropriate
● often appropriate
● very often appropriate
● usually appropriate
● almost always appropriate
● always appropriate

12) Hospitalization in case of acute asthma attack

a. is always indicated
● never appropriate
● hardly ever appropriate
● usually not appropriate
● sometimes appropriate
● often appropriate
● very often appropriate
● usually appropriate
● almost always appropriate
● always appropriate

b. is indicated if there is need for oxygen therapy
● never appropriate
● hardly ever appropriate
● usually not appropriate
● sometimes appropriate
● often appropriate
● very often appropriate
● usually appropriate
● almost always appropriate
● always appropriate

c. is indicated if patient is <6 years of age
● never appropriate

- hardly ever appropriate
- usually not appropriate
- sometimes appropriate
- often appropriate
- very often appropriate
- usually appropriate
- almost always appropriate
- always appropriate

d. is indicated if the patient is <1 year old
- never appropriate
- hardly ever appropriate
- usually not appropriate
- sometimes appropriate
- often appropriate
- very often appropriate
- usually appropriate
- almost always appropriate
- always appropriate

e. is always indicated if pneumonia is associated
- never appropriate
- hardly ever appropriate
- usually not appropriate
- sometimes appropriate
- often appropriate
- very often appropriate
- usually appropriate
- almost always appropriate
- always appropriate

f. is always indicated if pneumothorax is associated
- never appropriate
- hardly ever appropriate
- usually not appropriate
- sometimes appropriate
- often appropriate
- very often appropriate
- usually appropriate
- almost always appropriate
- always appropriate

g. is indicated if the patient has a history of a previous severe asthma attack that required access to PICU
- never appropriate
- hardly ever appropriate
- usually not appropriate
- sometimes appropriate
- often appropriate
- very often appropriate
- usually appropriate

- almost always appropriate
- always appropriate

h. is indicated if the patient has underlying diseases and pulmonary or extrapulmonary risk factors (e.g. obesity, psychiatric disorders, prematurity, pulmonary bronchodysplasia, bronchiectasis, congenital malformations involving the airways)
- never appropriate
- hardly ever appropriate
- usually not appropriate
- sometimes appropriate
- often appropriate
- very often appropriate
- usually appropriate
- almost always appropriate
- always appropriate

i. is indicated in case of poor family compliance
- never appropriate
- hardly ever appropriate
- usually not appropriate
- sometimes appropriate
- often appropriate
- very often appropriate
- usually appropriate
- almost always appropriate
- always appropriate

13) Following an episode of acute asthma attack

a. a specialist evaluation is always indicated
- never appropriate
- hardly ever appropriate
- usually not appropriate
- sometimes appropriate
- often appropriate
- very often appropriate
- usually appropriate
- almost always appropriate
- always appropriate

b. a specialist evaluation is indicated if the acute attack required hospitalization
- never appropriate
- hardly ever appropriate
- usually not appropriate
- sometimes appropriate
- often appropriate
- very often appropriate
- usually appropriate
- almost always appropriate

● always appropriate

c. specialist evaluation is indicated if the patient has underlying diseases and pulmonary or extrapulmonary risk factors (e.g. obesity, psychiatric disorders, prematurity, pulmonary bronchodysplasia, bronchiectasis, congenital malformations involving the airways)
● never appropriate
● hardly ever appropriate
● usually not appropriate
● sometimes appropriate
● often appropriate
● very often appropriate
● usually appropriate
● almost always appropriate
● always appropriate

d. spirometry is recommended (if the child is >5-6 years old)
● never appropriate
● hardly ever appropriate
● usually not appropriate
● sometimes appropriate
● often appropriate
● very often appropriate
● usually appropriate
● almost always appropriate
● always appropriate

e. the execution of prick skin test is always indicated
● never appropriate
● hardly ever appropriate
● usually not appropriate
● sometimes appropriate
● often appropriate
● very often appropriate
● usually appropriate
● almost always appropriate
● always appropriate

f. prick skin tests are recommended after 2 years of age
● never appropriate
● hardly ever appropriate
● usually not appropriate
● sometimes appropriate
● often appropriate
● very often appropriate
● usually appropriate
● almost always appropriate
● always appropriate

g. dosing total and specific IgE for inhaled allergens is indicated
● never appropriate
● hardly ever appropriate
● usually not appropriate

- sometimes appropriate
- often appropriate
- very often appropriate
- usually appropriate
- almost always appropriate
- always appropriate

h. the execution of allergy tests (prick test, specific IgE for inhaled allergens) is indicated only if there is a family history of allergy
- never appropriate
- hardly ever appropriate
- usually not appropriate
- sometimes appropriate
- often appropriate
- very often appropriate
- usually appropriate
- almost always appropriate
- always appropriate

i. the measurement of fractional exhaled nitric oxide (FeNO) is indicated
- never appropriate
- hardly ever appropriate
- usually not appropriate
- sometimes appropriate
- often appropriate
- very often appropriate
- usually appropriate
- almost always appropriate
- always appropriate
