# Supplementary material for: Management of Children with Acute Asthma Attack: A RAND/UCLA Appropriateness Approach
Source: Int J Environ Res Public Health. 2021 Dec 3;18(23):12775. doi: 10.3390/ijerph182312775 (PMC8657661; doi:10.3390/ijerph182312775)
Supplement: Supplementary file 1 [file ijerph-18-12775-s001.zip › Suppl_2_Graphs_PRE POST v1.pptx]

## Slide 1
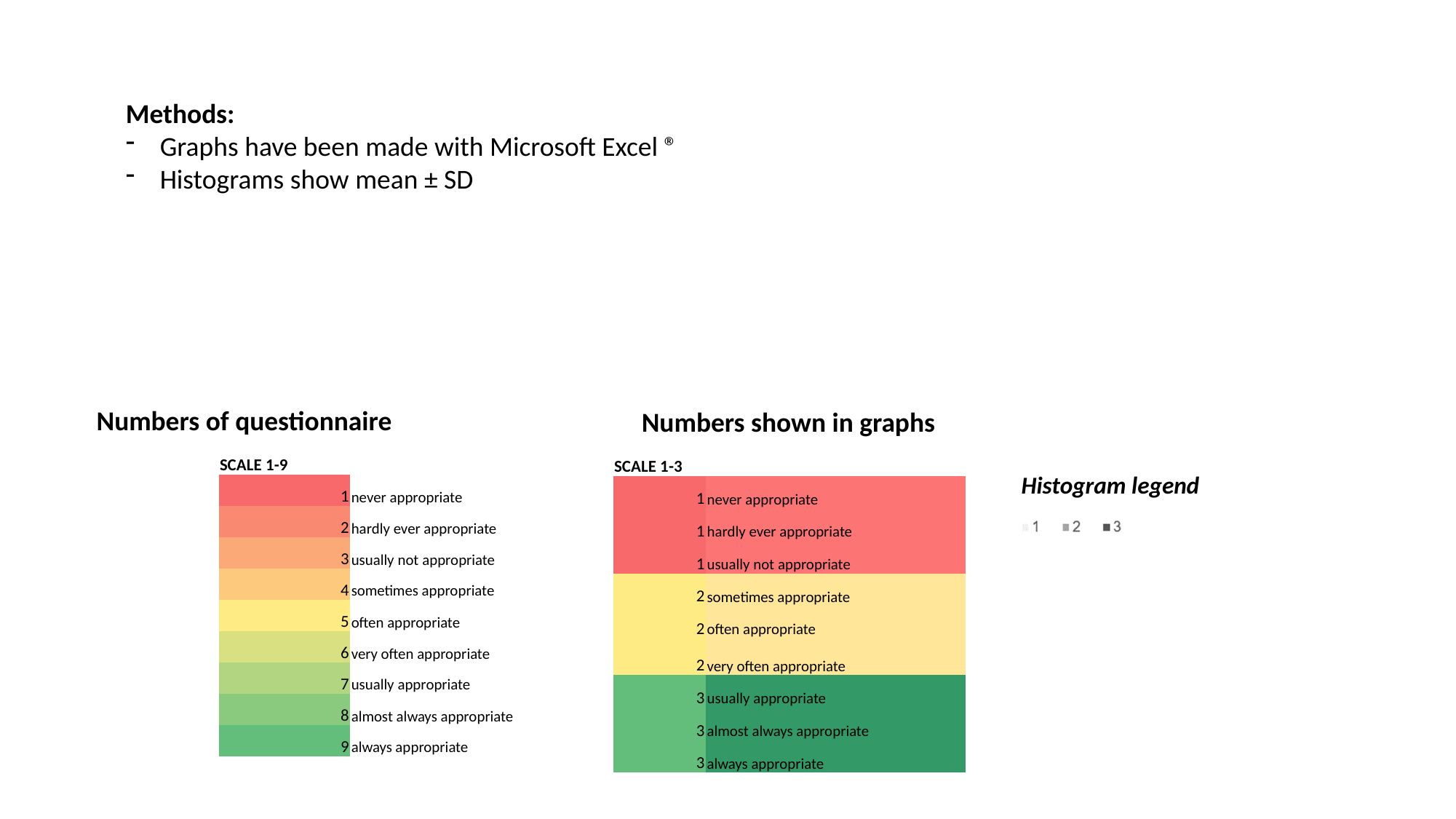

Methods:
Graphs have been made with Microsoft Excel ®
Histograms show mean ± SD
Numbers of questionnaire
Numbers shown in graphs
| SCALE 1-9 | |
| --- | --- |
| 1 | never appropriate |
| 2 | hardly ever appropriate |
| 3 | usually not appropriate |
| 4 | sometimes appropriate |
| 5 | often appropriate |
| 6 | very often appropriate |
| 7 | usually appropriate |
| 8 | almost always appropriate |
| 9 | always appropriate |
| SCALE 1-3 | |
| --- | --- |
| 1 | never appropriate |
| 1 | hardly ever appropriate |
| 1 | usually not appropriate |
| 2 | sometimes appropriate |
| 2 | often appropriate |
| 2 | very often appropriate |
| 3 | usually appropriate |
| 3 | almost always appropriate |
| 3 | always appropriate |
Histogram legend

## Slide 2
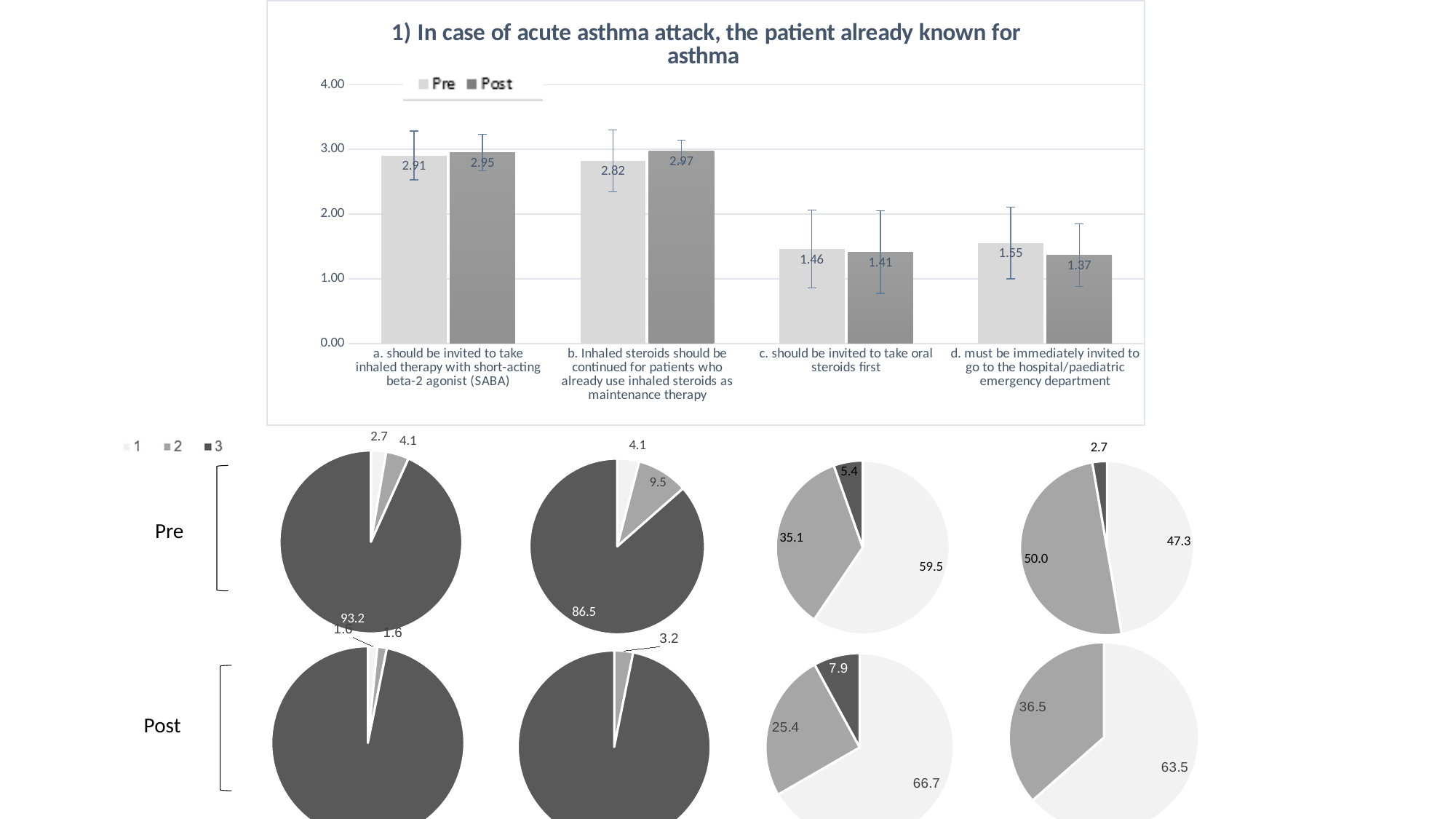

### Chart: 1) In case of acute asthma attack, the patient already known for asthma
| Category | | |
|---|---|---|
| a. should be invited to take inhaled therapy with short-acting beta-2 agonist (SABA) | 2.9054054 | 2.952380952380952 |
| b. Inhaled steroids should be continued for patients who already use inhaled steroids as maintenance therapy | 2.8243243 | 2.968253968253968 |
| c. should be invited to take oral steroids first | 1.4594595 | 1.412698412698413 |
| d. must be immediately invited to go to the hospital/paediatric emergency department | 1.5540541 | 1.365079365079365 |
### Chart
| Category | |
|---|---|
### Chart
| Category | |
|---|---|
### Chart
| Category | |
|---|---|
### Chart
| Category | |
|---|---|
Pre
### Chart
| Category | |
|---|---|
### Chart
| Category | |
|---|---|
### Chart
| Category | |
|---|---|
### Chart
| Category | |
|---|---|
Post

## Slide 3
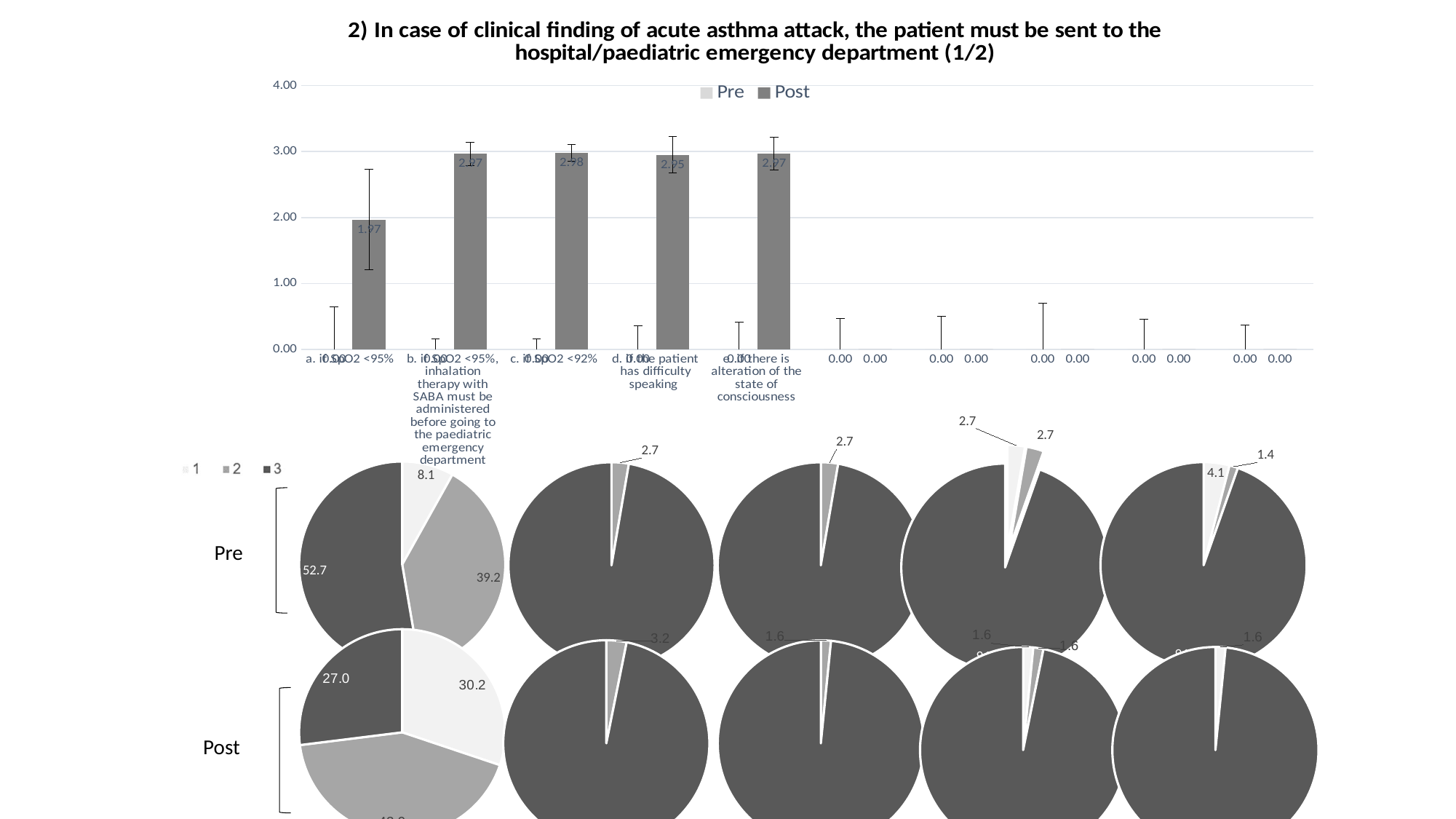

### Chart: 2) In case of clinical finding of acute asthma attack, the patient must be sent to the hospital/paediatric emergency department (1/2)
| Category | | |
|---|---|---|
| a. if SpO2 <95% | 2.4459459 | 1.968253968253968 |
| b. if SpO2 <95%, inhalation therapy with SABA must be administered before going to the paediatric emergency department | 2.972973 | 2.968253968253968 |
| c. if SpO2 <92% | 2.972973 | 2.984126984126984 |
| d. if the patient has difficulty speaking | 2.9189189 | 2.952380952380952 |
| e. if there is alteration of the state of consciousness | 2.9054054 | 2.968253968253968 |
### Chart
| Category | |
|---|---|
### Chart
| Category | |
|---|---|
### Chart
| Category | |
|---|---|
### Chart
| Category | |
|---|---|
### Chart
| Category | |
|---|---|
Pre
### Chart
| Category | |
|---|---|
### Chart
| Category | |
|---|---|
### Chart
| Category | |
|---|---|
### Chart
| Category | |
|---|---|
### Chart
| Category | |
|---|---|
Post

## Slide 4
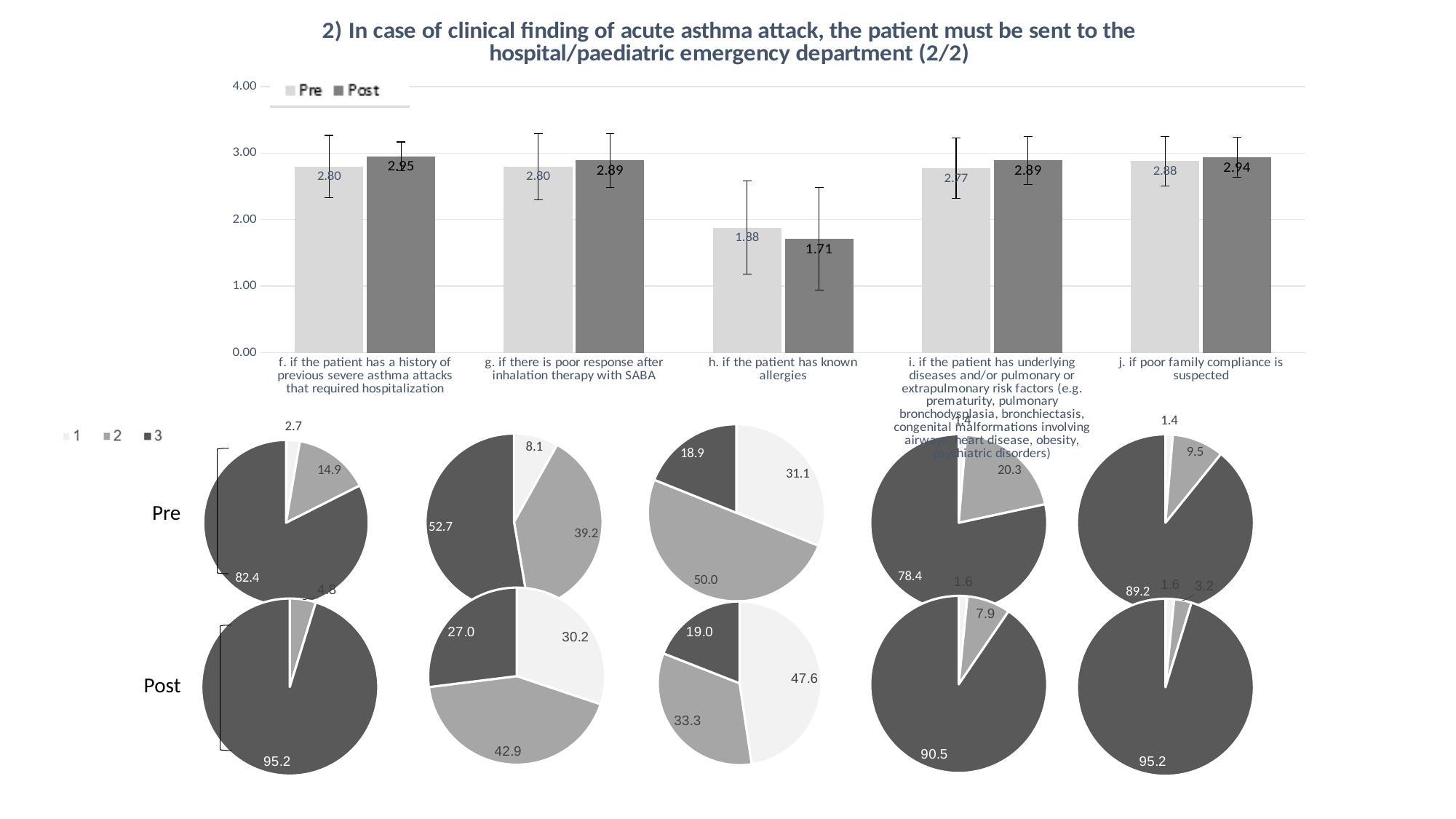

### Chart: 2) In case of clinical finding of acute asthma attack, the patient must be sent to the hospital/paediatric emergency department (2/2)
| Category | | |
|---|---|---|
| f. if the patient has a history of previous severe asthma attacks that required hospitalization | 2.7972973 | 2.952380952380952 |
| g. if there is poor response after inhalation therapy with SABA | 2.7972973 | 2.888888888888889 |
| h. if the patient has known allergies | 1.8783784 | 1.714285714285714 |
| i. if the patient has underlying diseases and/or pulmonary or extrapulmonary risk factors (e.g. prematurity, pulmonary bronchodysplasia, bronchiectasis, congenital malformations involving airways, heart disease, obesity, psychiatric disorders) | 2.7702703 | 2.888888888888889 |
| j. if poor family compliance is suspected | 2.8783784 | 2.936507936507936 |
### Chart
| Category | |
|---|---|
### Chart
| Category | |
|---|---|
### Chart
| Category | |
|---|---|
### Chart
| Category | |
|---|---|
### Chart
| Category | |
|---|---|
Pre
### Chart
| Category | |
|---|---|
### Chart
| Category | |
|---|---|
### Chart
| Category | |
|---|---|
### Chart
| Category | |
|---|---|
### Chart
| Category | |
|---|---|
Post

## Slide 5
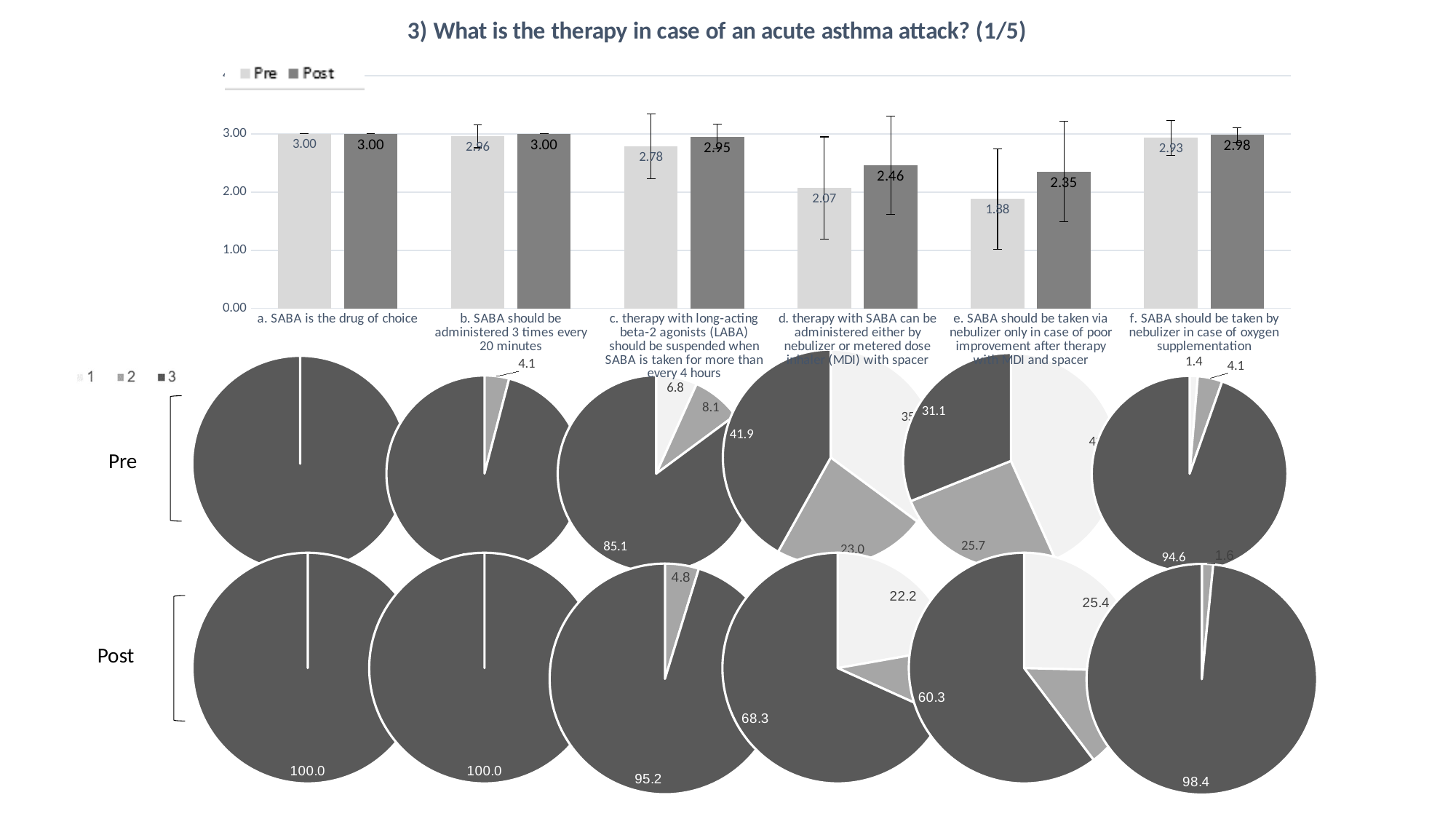

### Chart: 3) What is the therapy in case of an acute asthma attack? (1/5)
| Category | | |
|---|---|---|
| a. SABA is the drug of choice | 3.0 | 3.0 |
| b. SABA should be administered 3 times every 20 minutes | 2.9594595 | 3.0 |
| c. therapy with long-acting beta-2 agonists (LABA) should be suspended when SABA is taken for more than every 4 hours | 2.7837838 | 2.952380952380952 |
| d. therapy with SABA can be administered either by nebulizer or metered dose inhaler (MDI) with spacer | 2.0675676 | 2.46031746031746 |
| e. SABA should be taken via nebulizer only in case of poor improvement after therapy with MDI and spacer | 1.8783784 | 2.349206349206349 |
| f. SABA should be taken by nebulizer in case of oxygen supplementation | 2.9324324 | 2.984126984126984 |
### Chart
| Category | |
|---|---|
### Chart
| Category | |
|---|---|
### Chart
| Category | |
|---|---|
### Chart
| Category | |
|---|---|
### Chart
| Category | |
|---|---|
### Chart
| Category | |
|---|---|
Pre
### Chart
| Category | |
|---|---|
### Chart
| Category | |
|---|---|
### Chart
| Category | |
|---|---|
### Chart
| Category | |
|---|---|
### Chart
| Category | |
|---|---|
### Chart
| Category | |
|---|---|
Post

## Slide 6
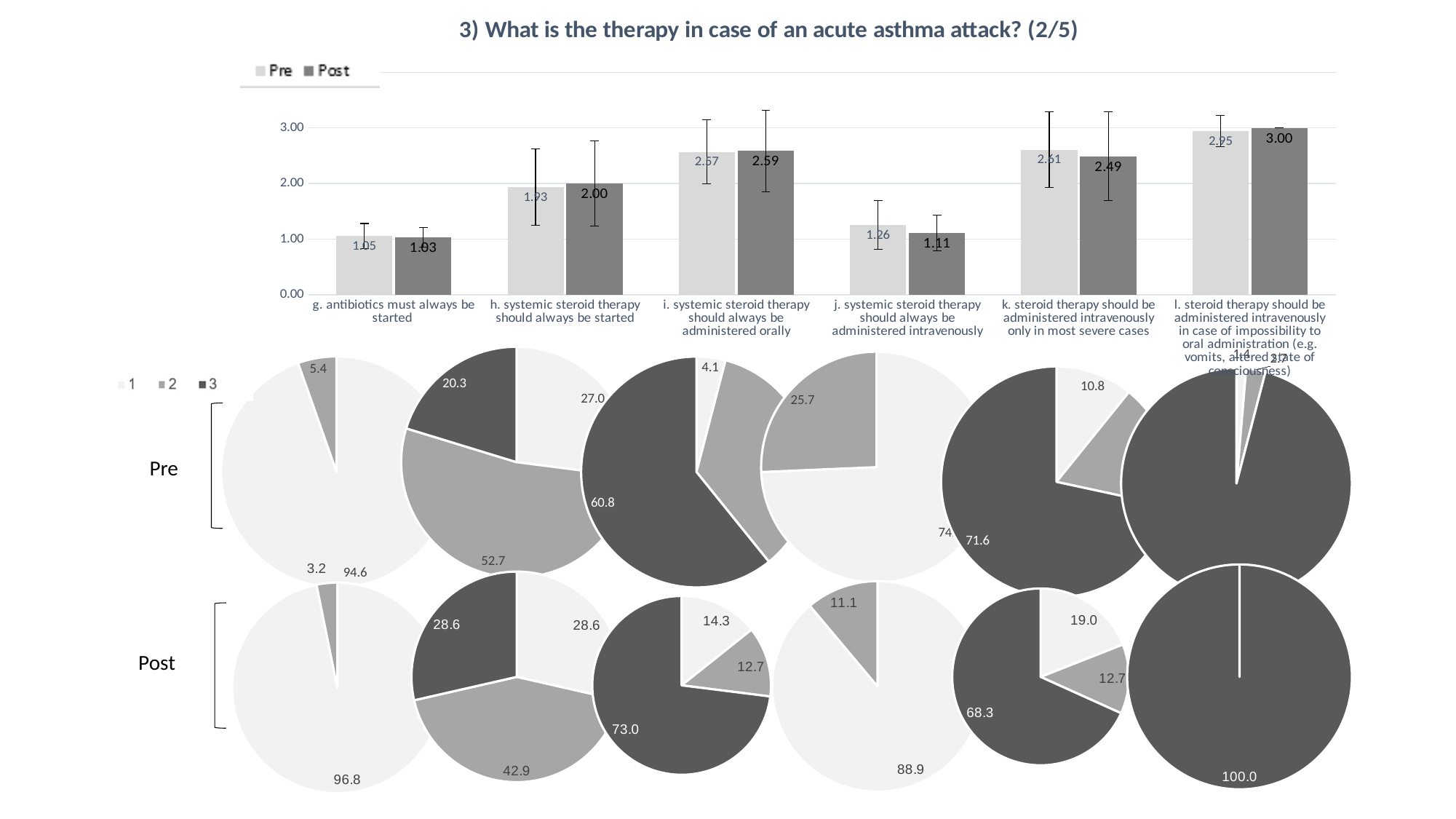

### Chart: 3) What is the therapy in case of an acute asthma attack? (2/5)
| Category | | |
|---|---|---|
| g. antibiotics must always be started | 1.0540541 | 1.031746031746032 |
| h. systemic steroid therapy should always be started | 1.9324324 | 2.0 |
| i. systemic steroid therapy should always be administered orally | 2.5675676 | 2.587301587301587 |
| j. systemic steroid therapy should always be administered intravenously | 1.2567568 | 1.111111111111111 |
| k. steroid therapy should be administered intravenously only in most severe cases | 2.6081081 | 2.492063492063492 |
| l. steroid therapy should be administered intravenously in case of impossibility to oral administration (e.g. vomits, altered state of consciousness) | 2.9459459 | 3.0 |
### Chart
| Category | |
|---|---|
### Chart
| Category | |
|---|---|
### Chart
| Category | |
|---|---|
### Chart
| Category | |
|---|---|
### Chart
| Category | |
|---|---|
### Chart
| Category | |
|---|---|
Pre
### Chart
| Category | |
|---|---|
### Chart
| Category | |
|---|---|
### Chart
| Category | |
|---|---|
### Chart
| Category | |
|---|---|
### Chart
| Category | |
|---|---|
### Chart
| Category | |
|---|---|
Post

## Slide 7
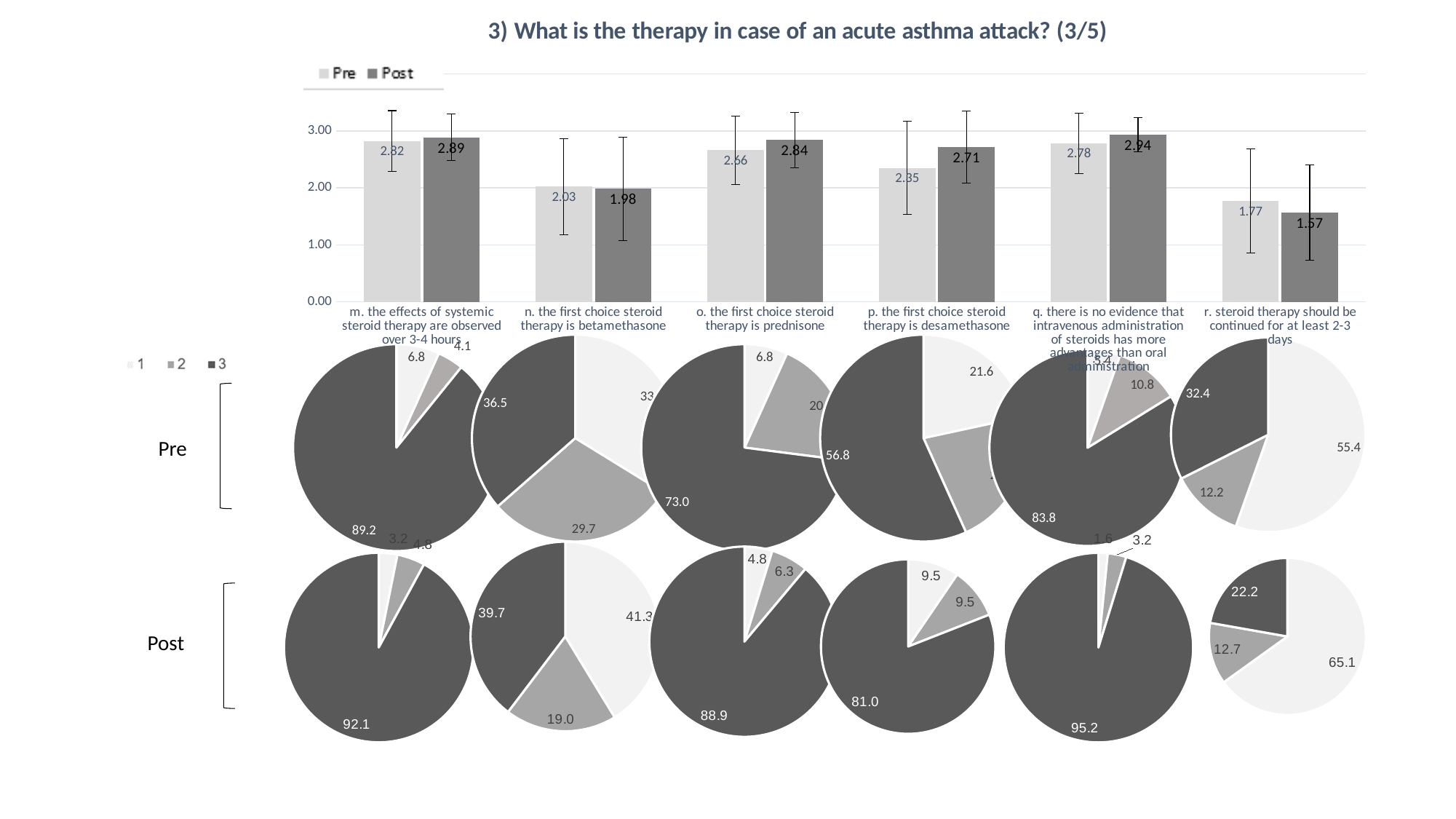

### Chart: 3) What is the therapy in case of an acute asthma attack? (3/5)
| Category | | |
|---|---|---|
| m. the effects of systemic steroid therapy are observed over 3-4 hours | 2.8243243 | 2.888888888888889 |
| n. the first choice steroid therapy is betamethasone | 2.027027 | 1.984126984126984 |
| o. the first choice steroid therapy is prednisone | 2.6621622 | 2.841269841269841 |
| p. the first choice steroid therapy is desamethasone | 2.3513514 | 2.714285714285714 |
| q. there is no evidence that intravenous administration of steroids has more advantages than oral administration | 2.7837838 | 2.936507936507936 |
| r. steroid therapy should be continued for at least 2-3 days | 1.7702703 | 1.571428571428571 |
### Chart
| Category | |
|---|---|
### Chart
| Category | |
|---|---|
### Chart
| Category | |
|---|---|
### Chart
| Category | |
|---|---|
### Chart
| Category | |
|---|---|
### Chart
| Category | |
|---|---|
Pre
### Chart
| Category | |
|---|---|
### Chart
| Category | |
|---|---|
### Chart
| Category | |
|---|---|
### Chart
| Category | |
|---|---|
### Chart
| Category | |
|---|---|
### Chart
| Category | |
|---|---|
Post

## Slide 8
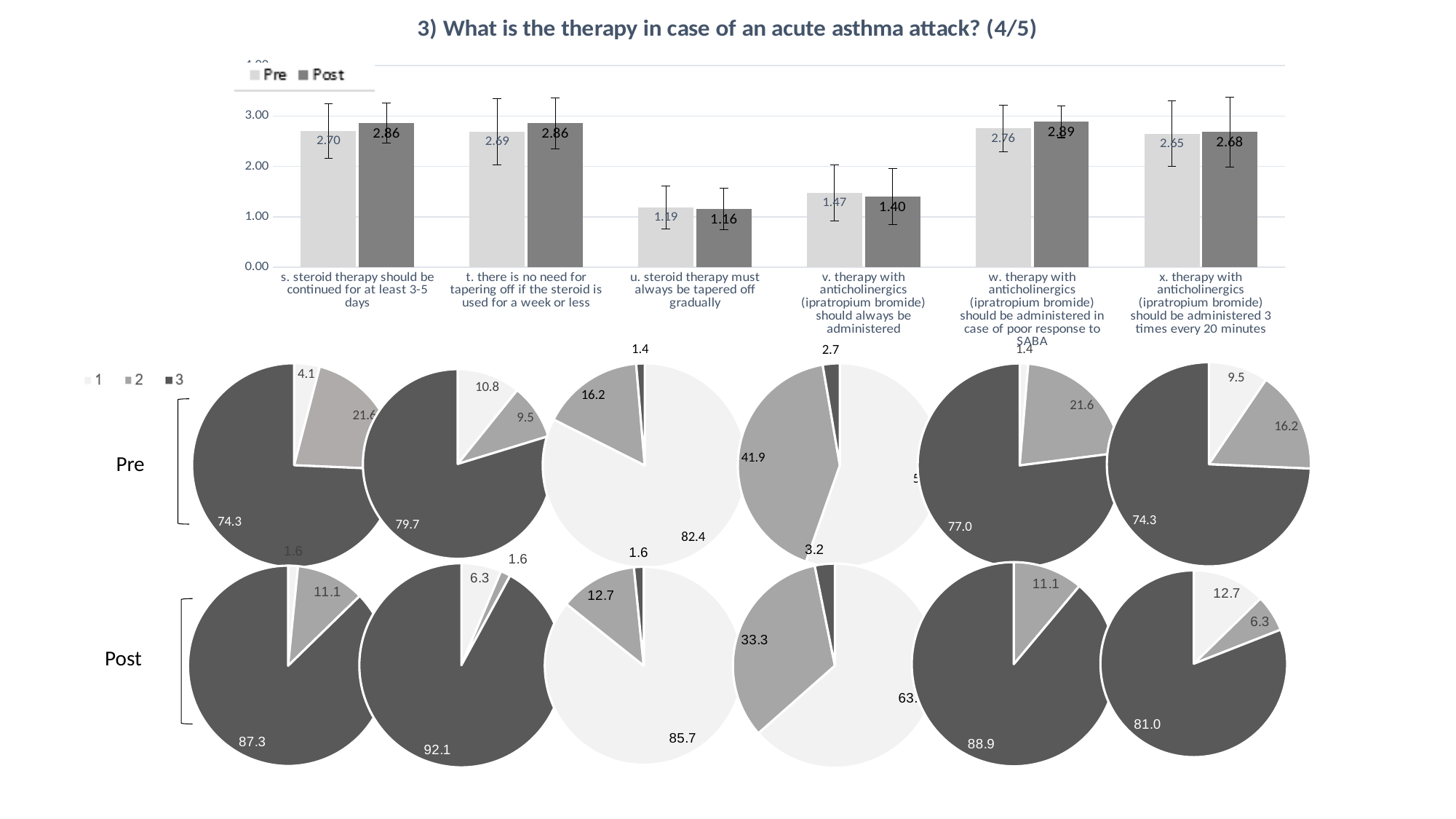

### Chart: 3) What is the therapy in case of an acute asthma attack? (4/5)
| Category | | |
|---|---|---|
| s. steroid therapy should be continued for at least 3-5 days | 2.7027027 | 2.857142857142857 |
| t. there is no need for tapering off if the steroid is used for a week or less | 2.6891892 | 2.857142857142857 |
| u. steroid therapy must always be tapered off gradually | 1.1891892 | 1.158730158730159 |
| v. therapy with anticholinergics (ipratropium bromide) should always be administered | 1.472973 | 1.396825396825397 |
| w. therapy with anticholinergics (ipratropium bromide) should be administered in case of poor response to SABA | 2.7567568 | 2.888888888888889 |
| x. therapy with anticholinergics (ipratropium bromide) should be administered 3 times every 20 minutes | 2.6486486 | 2.682539682539682 |
### Chart
| Category | |
|---|---|
### Chart
| Category | |
|---|---|
### Chart
| Category | |
|---|---|
### Chart
| Category | |
|---|---|
### Chart
| Category | |
|---|---|
### Chart
| Category | |
|---|---|
Pre
### Chart
| Category | |
|---|---|
### Chart
| Category | |
|---|---|
### Chart
| Category | |
|---|---|
### Chart
| Category | |
|---|---|
### Chart
| Category | |
|---|---|
### Chart
| Category | |
|---|---|
Post

## Slide 9
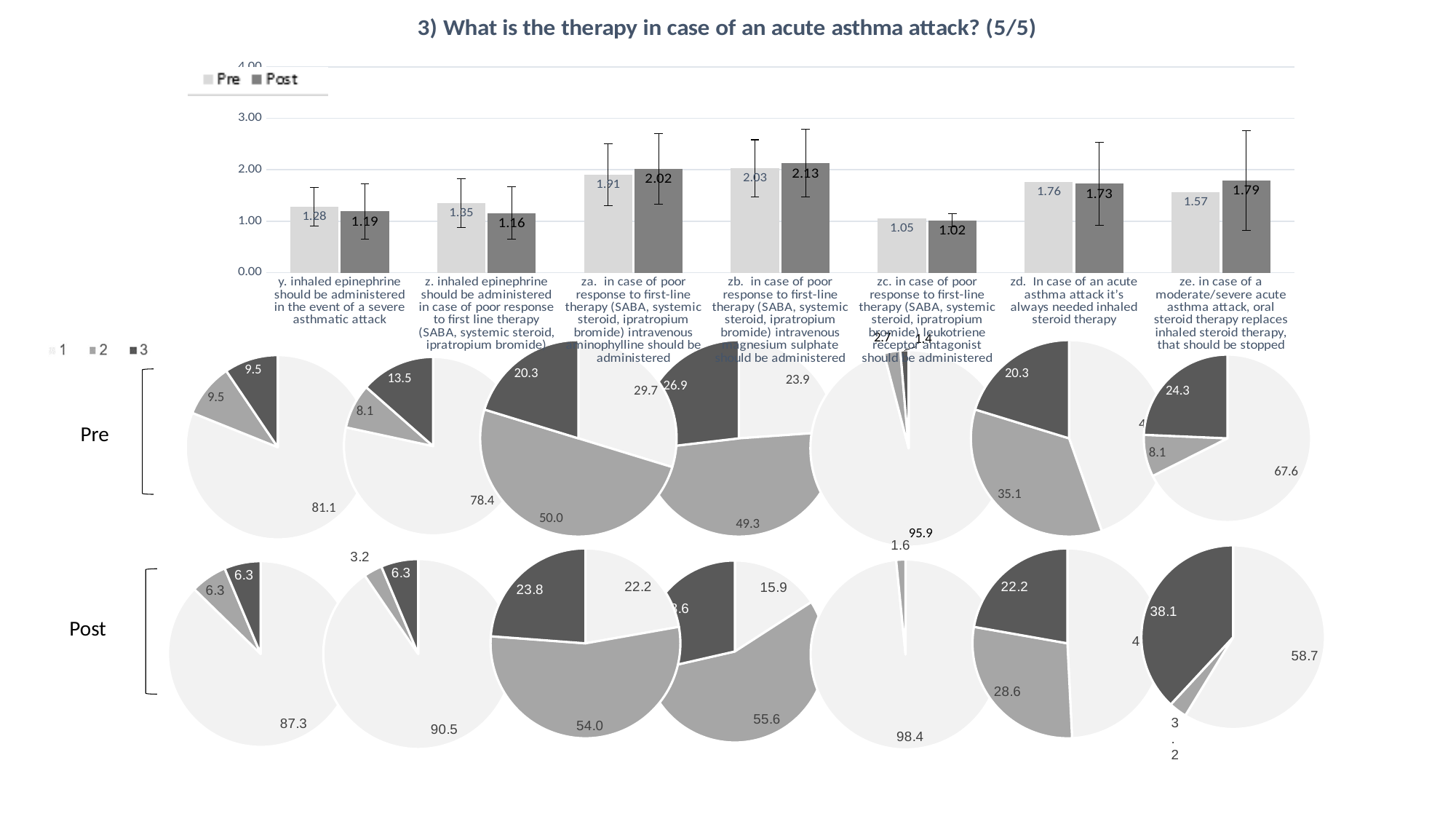

### Chart: 3) What is the therapy in case of an acute asthma attack? (5/5)
| Category | | |
|---|---|---|
| y. inhaled epinephrine should be administered in the event of a severe asthmatic attack | 1.2837838 | 1.19047619047619 |
| z. inhaled epinephrine should be administered in case of poor response to first line therapy (SABA, systemic steroid, ipratropium bromide) | 1.3513514 | 1.158730158730159 |
| za. in case of poor response to first-line therapy (SABA, systemic steroid, ipratropium bromide) intravenous aminophylline should be administered | 1.9054054 | 2.015873015873016 |
| zb. in case of poor response to first-line therapy (SABA, systemic steroid, ipratropium bromide) intravenous magnesium sulphate should be administered | 2.0298507 | 2.126984126984127 |
| zc. in case of poor response to first-line therapy (SABA, systemic steroid, ipratropium bromide) leukotriene receptor antagonist should be administered | 1.0540541 | 1.015873015873016 |
| zd. In case of an acute asthma attack it’s always needed inhaled steroid therapy | 1.7567568 | 1.73015873015873 |
| ze. in case of a moderate/severe acute asthma attack, oral steroid therapy replaces inhaled steroid therapy, that should be stopped | 1.5675676 | 1.793650793650794 |
### Chart
| Category | |
|---|---|
### Chart
| Category | |
|---|---|
### Chart
| Category | |
|---|---|
### Chart
| Category | |
|---|---|
### Chart
| Category | |
|---|---|
### Chart
| Category | |
|---|---|
### Chart
| Category | |
|---|---|
Pre
### Chart
| Category | |
|---|---|
### Chart
| Category | |
|---|---|
### Chart
| Category | |
|---|---|
### Chart
| Category | |
|---|---|
### Chart
| Category | |
|---|---|
### Chart
| Category | |
|---|---|
### Chart
| Category | |
|---|---|
Post

## Slide 10
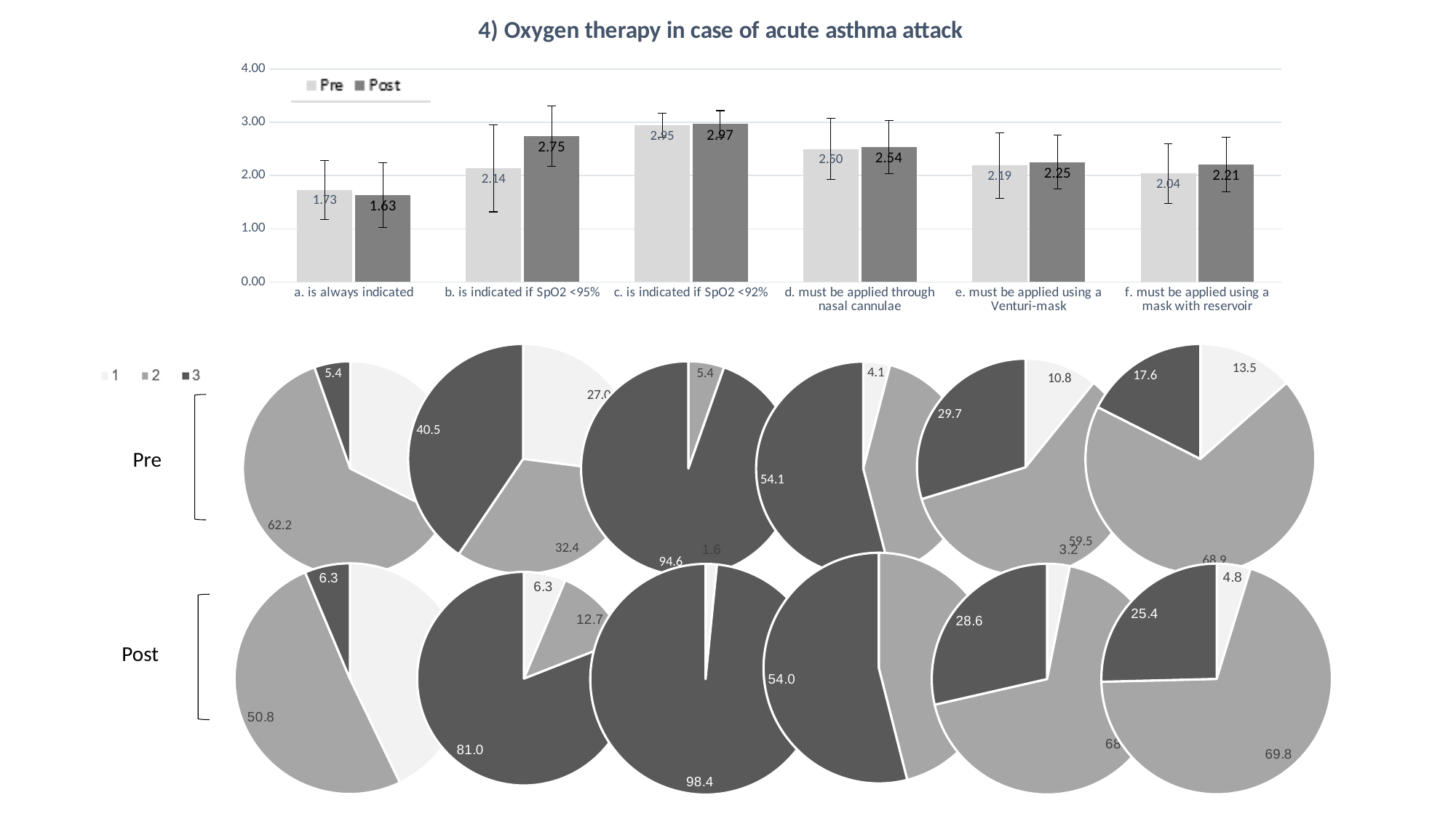

### Chart: 4) Oxygen therapy in case of acute asthma attack
| Category | | |
|---|---|---|
| a. is always indicated | 1.7297297 | 1.634920634920635 |
| b. is indicated if SpO2 <95% | 2.1351351 | 2.746031746031746 |
| c. is indicated if SpO2 <92% | 2.9459459 | 2.968253968253968 |
| d. must be applied through nasal cannulae | 2.5 | 2.53968253968254 |
| e. must be applied using a Venturi-mask | 2.1891892 | 2.253968253968254 |
| f. must be applied using a mask with reservoir | 2.0405405 | 2.206349206349206 |
### Chart
| Category | |
|---|---|
### Chart
| Category | |
|---|---|
### Chart
| Category | |
|---|---|
### Chart
| Category | |
|---|---|
### Chart
| Category | |
|---|---|
### Chart
| Category | |
|---|---|
Pre
### Chart
| Category | |
|---|---|
### Chart
| Category | |
|---|---|
### Chart
| Category | |
|---|---|
### Chart
| Category | |
|---|---|
### Chart
| Category | |
|---|---|
### Chart
| Category | |
|---|---|
Post

## Slide 11
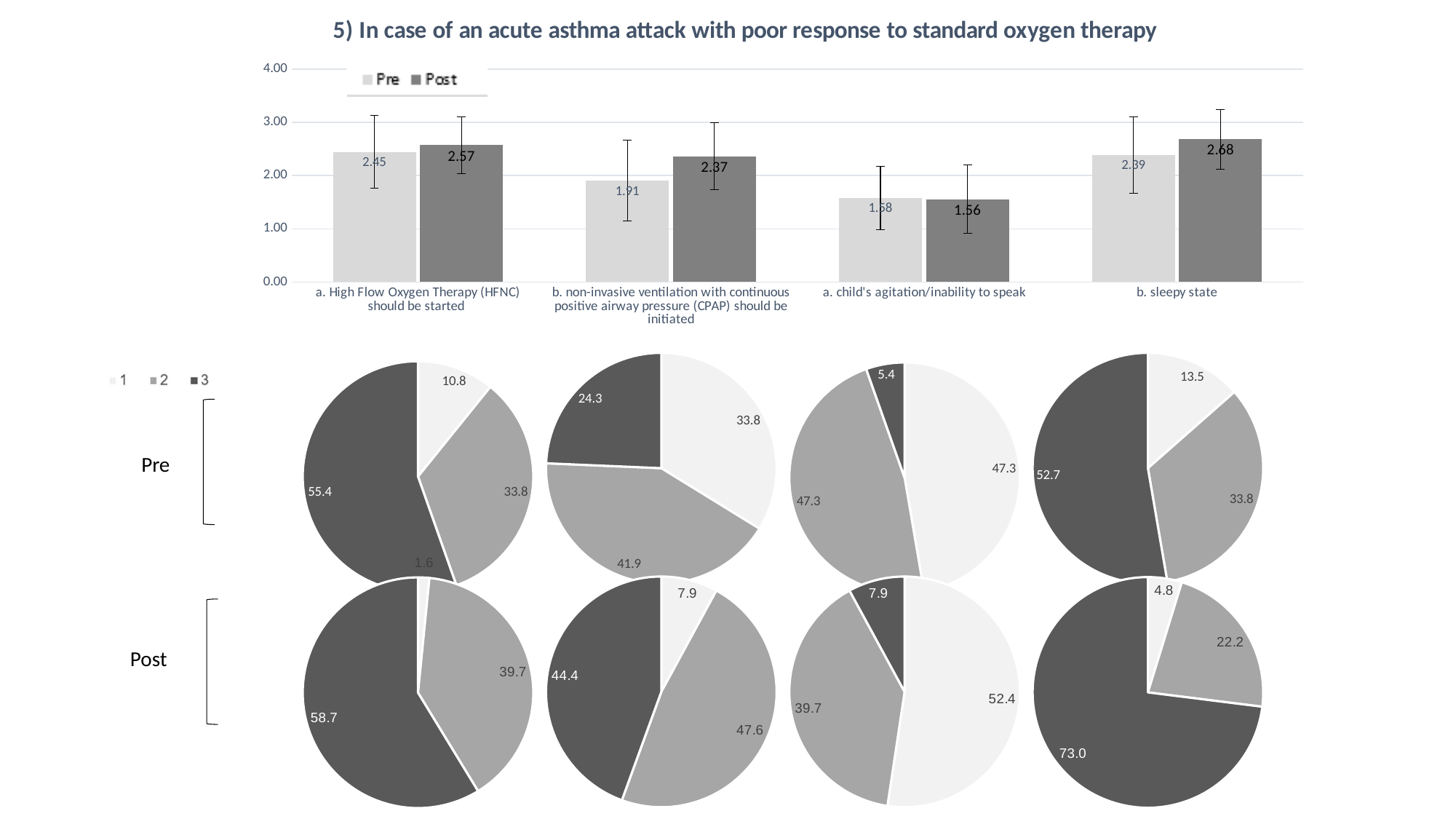

### Chart: 5) In case of an acute asthma attack with poor response to standard oxygen therapy
| Category | | |
|---|---|---|
| a. High Flow Oxygen Therapy (HFNC) should be started | 2.4459459 | 2.571428571428572 |
| b. non-invasive ventilation with continuous positive airway pressure (CPAP) should be initiated | 1.9054054 | 2.365079365079365 |
| a. child's agitation/inability to speak | 1.5810811 | 1.555555555555556 |
| b. sleepy state | 2.3918919 | 2.682539682539682 |
### Chart
| Category | |
|---|---|
### Chart
| Category | |
|---|---|
### Chart
| Category | |
|---|---|
### Chart
| Category | |
|---|---|
Pre
### Chart
| Category | |
|---|---|
### Chart
| Category | |
|---|---|
### Chart
| Category | |
|---|---|
### Chart
| Category | |
|---|---|
Post

## Slide 12
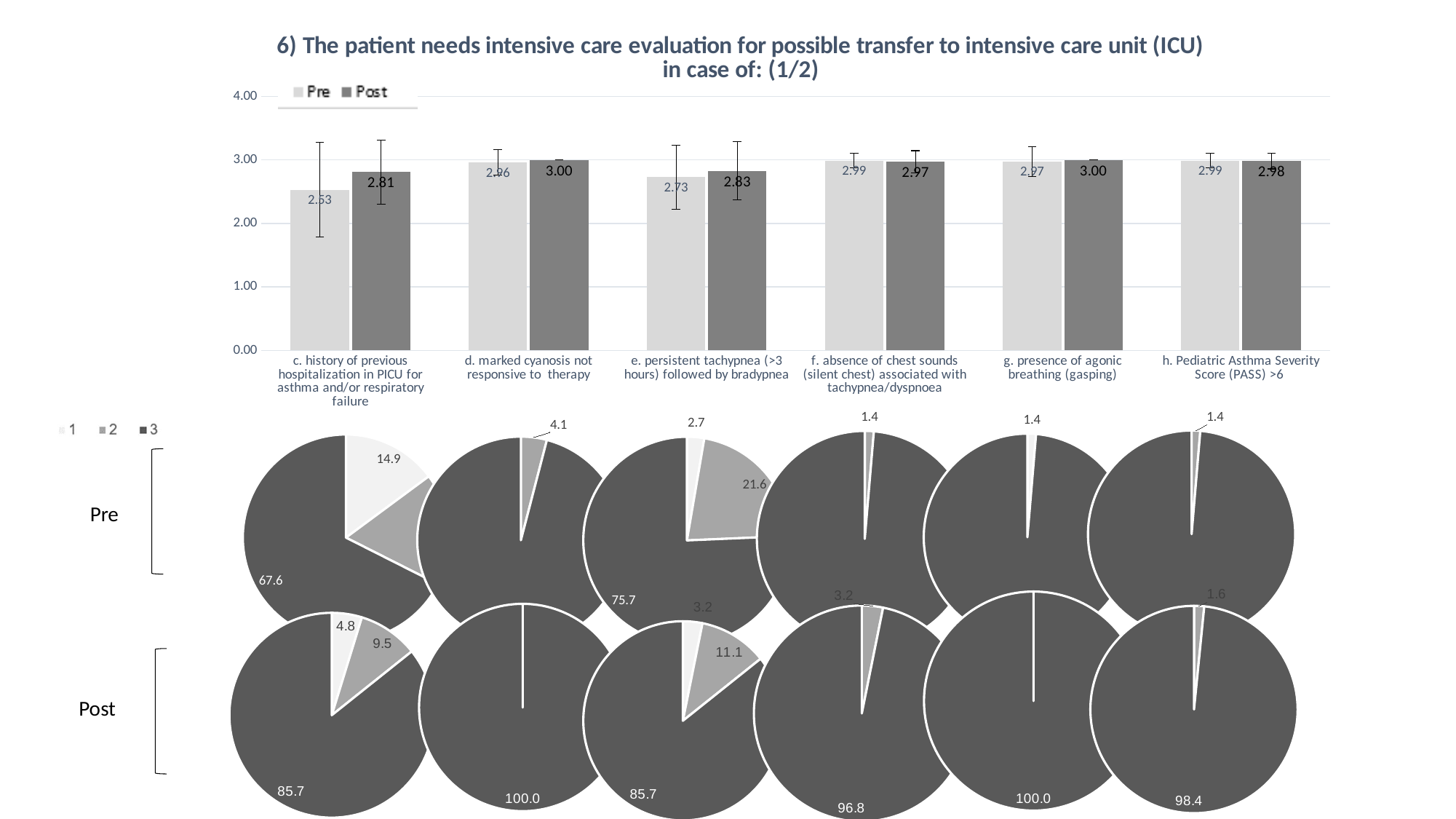

### Chart: 6) The patient needs intensive care evaluation for possible transfer to intensive care unit (ICU) in case of: (1/2)
| Category | | |
|---|---|---|
| c. history of previous hospitalization in PICU for asthma and/or respiratory failure | 2.527027 | 2.809523809523809 |
| d. marked cyanosis not responsive to therapy | 2.9594595 | 3.0 |
| e. persistent tachypnea (>3 hours) followed by bradypnea | 2.7297297 | 2.825396825396826 |
| f. absence of chest sounds (silent chest) associated with tachypnea/dyspnoea | 2.9864865 | 2.968253968253968 |
| g. presence of agonic breathing (gasping) | 2.972973 | 3.0 |
| h. Pediatric Asthma Severity Score (PASS) >6 | 2.9864865 | 2.984126984126984 |
### Chart
| Category | |
|---|---|
### Chart
| Category | |
|---|---|
### Chart
| Category | |
|---|---|
### Chart
| Category | |
|---|---|
### Chart
| Category | |
|---|---|
### Chart
| Category | |
|---|---|
Pre
### Chart
| Category | |
|---|---|
### Chart
| Category | |
|---|---|
### Chart
| Category | |
|---|---|
### Chart
| Category | |
|---|---|
### Chart
| Category | |
|---|---|
### Chart
| Category | |
|---|---|
Post

## Slide 13
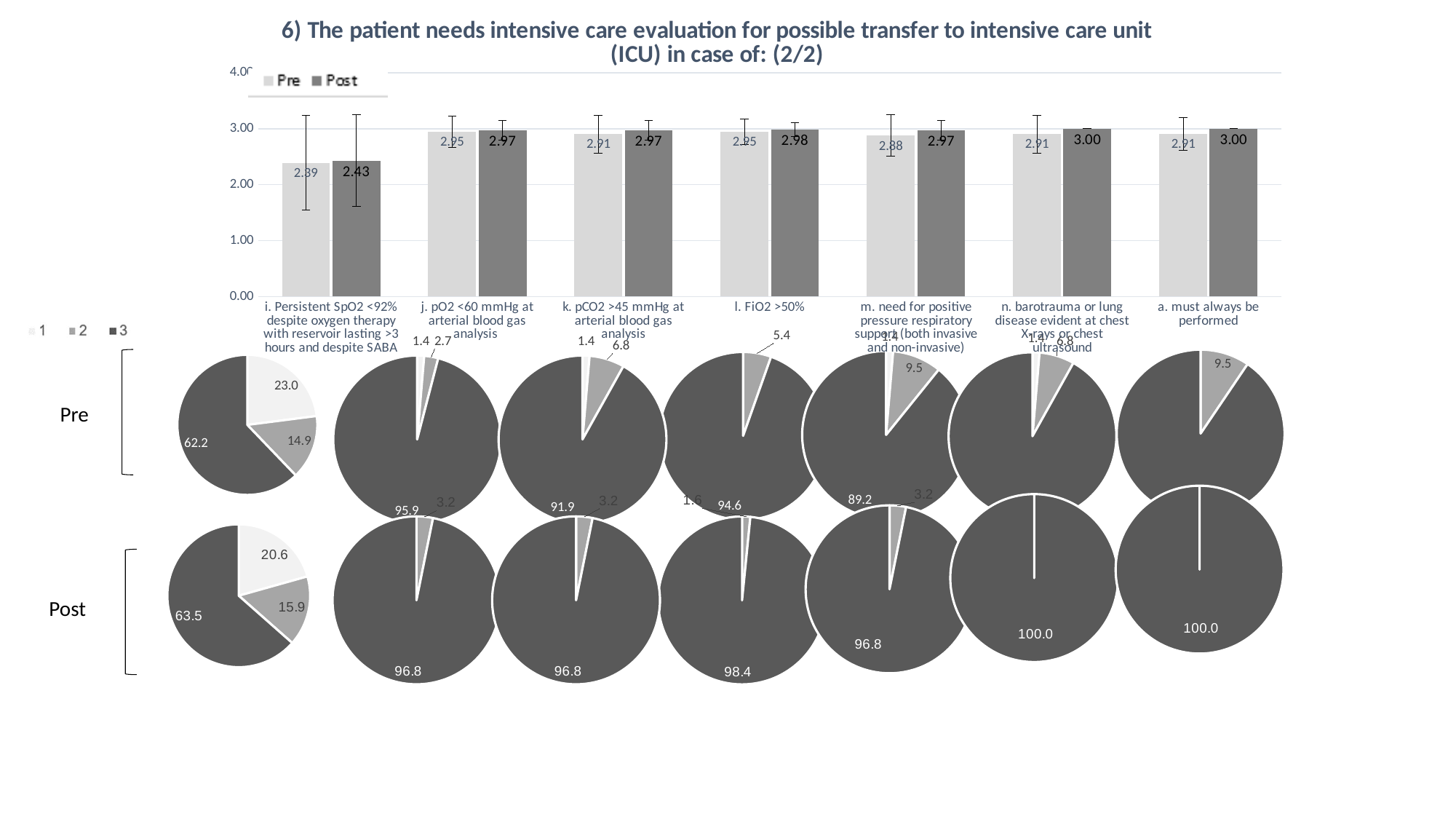

### Chart: 6) The patient needs intensive care evaluation for possible transfer to intensive care unit (ICU) in case of: (2/2)
| Category | | |
|---|---|---|
| i. Persistent SpO2 <92% despite oxygen therapy with reservoir lasting >3 hours and despite SABA | 2.3918919 | 2.428571428571428 |
| j. pO2 <60 mmHg at arterial blood gas analysis | 2.9459459 | 2.968253968253968 |
| k. pCO2 >45 mmHg at arterial blood gas analysis | 2.9054054 | 2.968253968253968 |
| l. FiO2 >50% | 2.9459459 | 2.984126984126984 |
| m. need for positive pressure respiratory support (both invasive and non-invasive) | 2.8783784 | 2.968253968253968 |
| n. barotrauma or lung disease evident at chest X-rays or chest ultrasound | 2.9054054 | 3.0 |
| a. must always be performed | 2.9054054 | 3.0 |
### Chart
| Category | |
|---|---|
### Chart
| Category | |
|---|---|
### Chart
| Category | |
|---|---|
### Chart
| Category | |
|---|---|
### Chart
| Category | |
|---|---|
### Chart
| Category | |
|---|---|
### Chart
| Category | |
|---|---|
Pre
### Chart
| Category | |
|---|---|
### Chart
| Category | |
|---|---|
### Chart
| Category | |
|---|---|
### Chart
| Category | |
|---|---|
### Chart
| Category | |
|---|---|
### Chart
| Category | |
|---|---|
### Chart
| Category | |
|---|---|
Post

## Slide 14
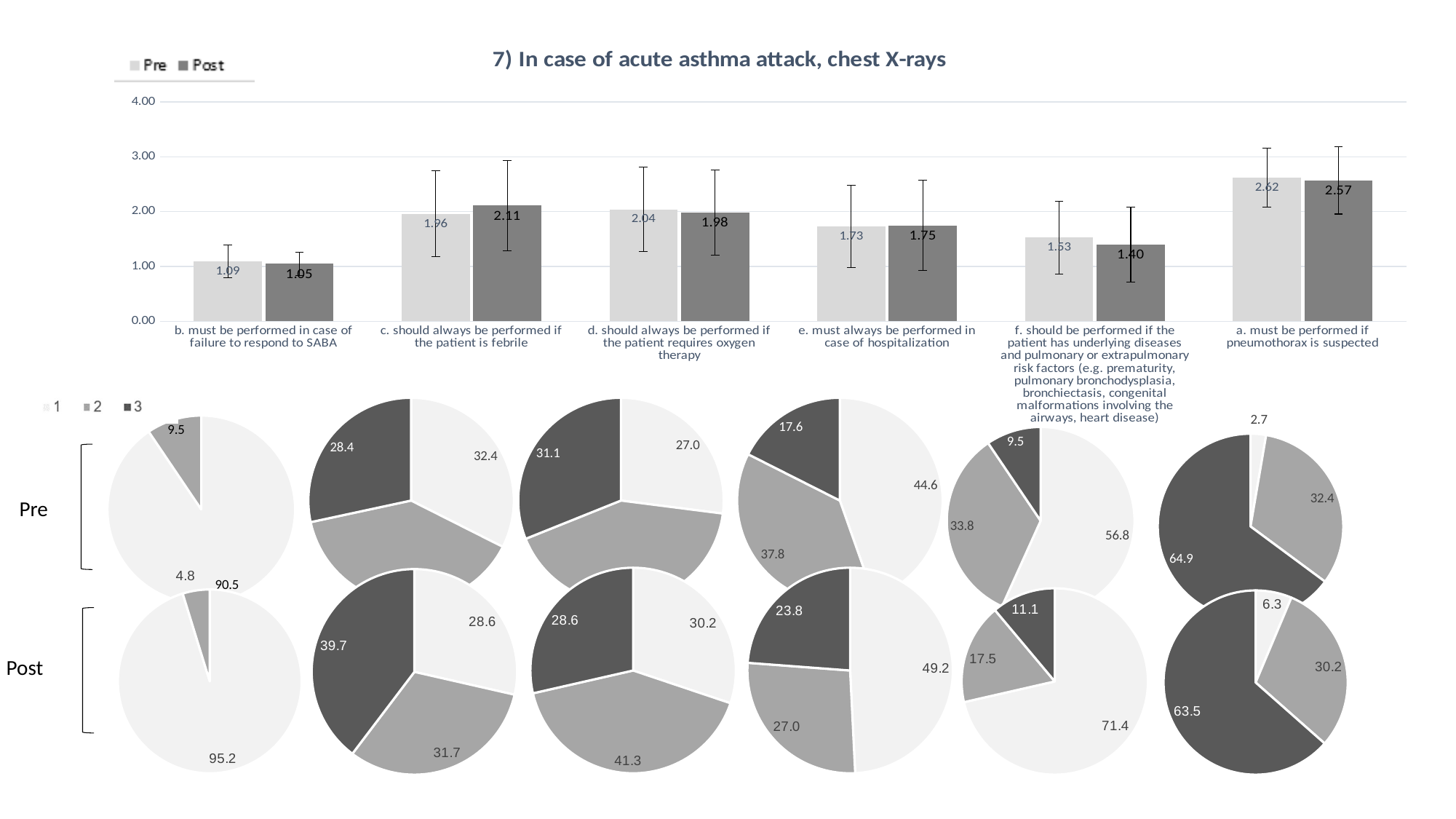

### Chart: 7) In case of acute asthma attack, chest X-rays
| Category | | |
|---|---|---|
| b. must be performed in case of failure to respond to SABA | 1.0945946 | 1.047619047619048 |
| c. should always be performed if the patient is febrile | 1.9594595 | 2.111111111111111 |
| d. should always be performed if the patient requires oxygen therapy | 2.0405405 | 1.984126984126984 |
| e. must always be performed in case of hospitalization | 1.7297297 | 1.746031746031746 |
| f. should be performed if the patient has underlying diseases and pulmonary or extrapulmonary risk factors (e.g. prematurity, pulmonary bronchodysplasia, bronchiectasis, congenital malformations involving the airways, heart disease) | 1.527027 | 1.396825396825397 |
| a. must be performed if pneumothorax is suspected | 2.6216216 | 2.571428571428572 |
### Chart
| Category | |
|---|---|
### Chart
| Category | |
|---|---|
### Chart
| Category | |
|---|---|
### Chart
| Category | |
|---|---|
### Chart
| Category | |
|---|---|
### Chart
| Category | |
|---|---|
Pre
### Chart
| Category | |
|---|---|
### Chart
| Category | |
|---|---|
### Chart
| Category | |
|---|---|
### Chart
| Category | |
|---|---|
### Chart
| Category | |
|---|---|
### Chart
| Category | |
|---|---|
Post

## Slide 15
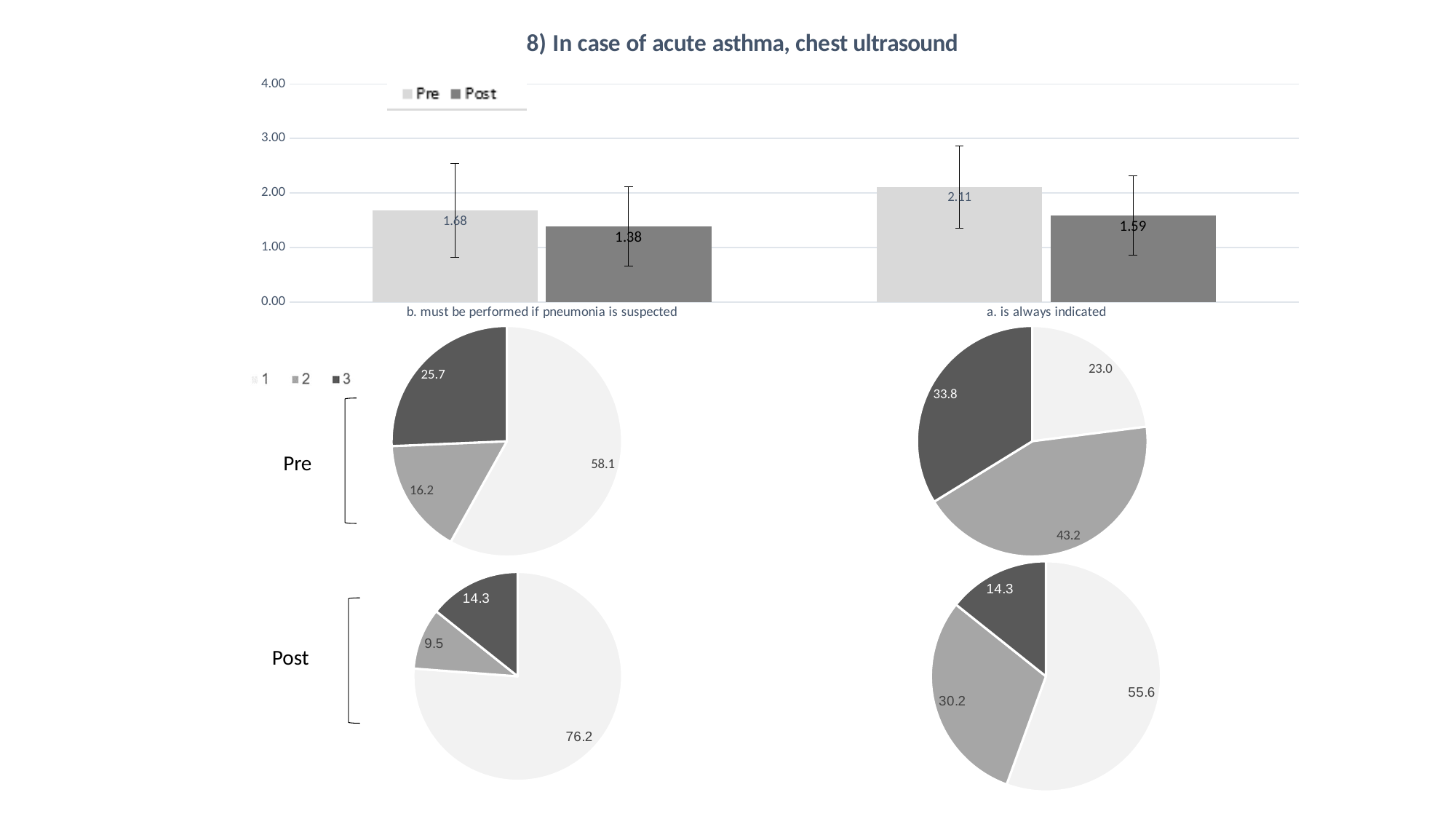

### Chart: 8) In case of acute asthma, chest ultrasound
| Category | | |
|---|---|---|
| b. must be performed if pneumonia is suspected | 1.6756757 | 1.380952380952381 |
| a. is always indicated | 2.1081081 | 1.587301587301587 |
### Chart
| Category | |
|---|---|
### Chart
| Category | |
|---|---|
Pre
### Chart
| Category | |
|---|---|
### Chart
| Category | |
|---|---|
Post

## Slide 16
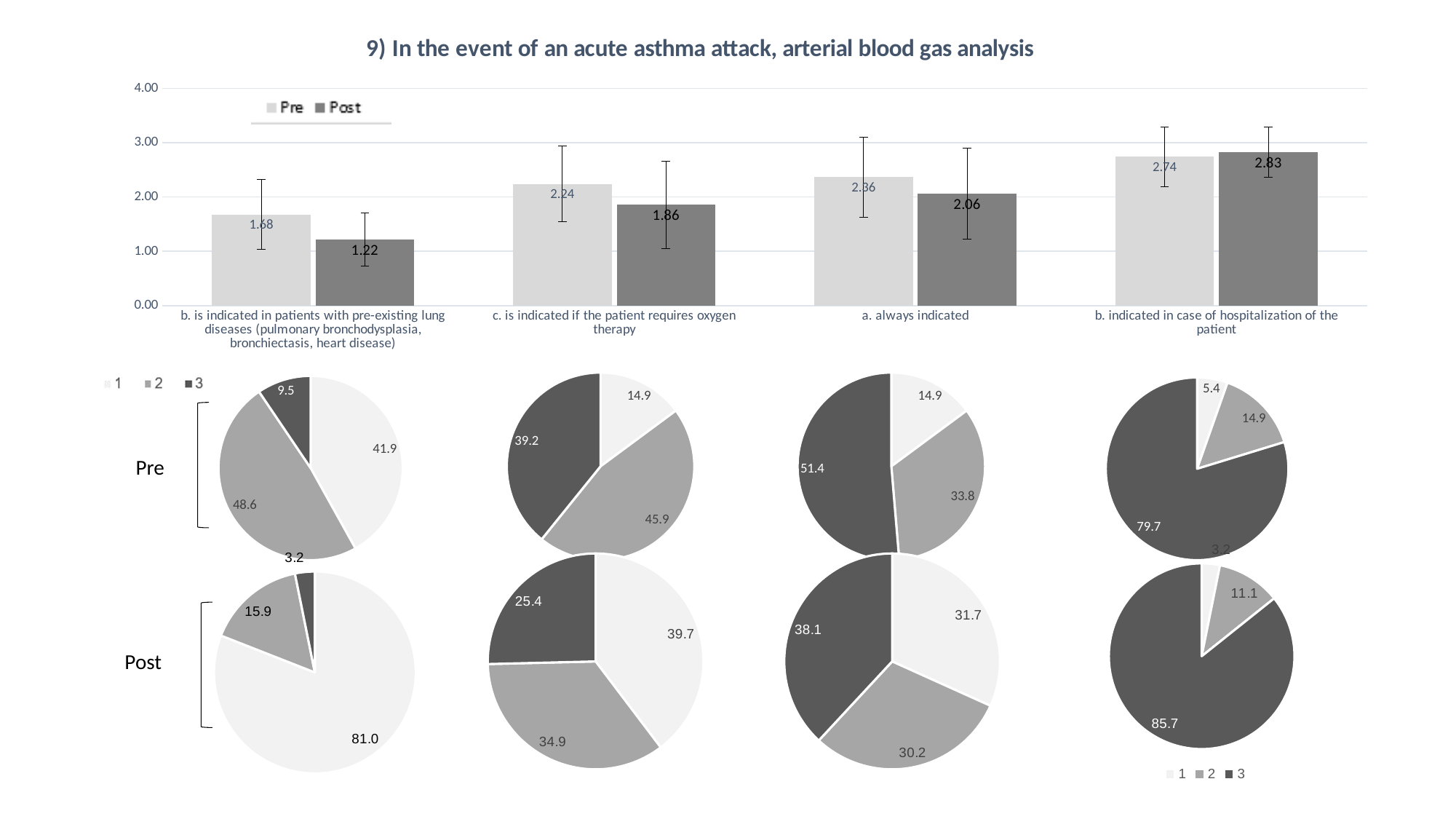

### Chart: 9) In the event of an acute asthma attack, arterial blood gas analysis
| Category | | |
|---|---|---|
| b. is indicated in patients with pre-existing lung diseases (pulmonary bronchodysplasia, bronchiectasis, heart disease) | 1.6756757 | 1.222222222222222 |
| c. is indicated if the patient requires oxygen therapy | 2.2432432 | 1.857142857142857 |
| a. always indicated | 2.3648649 | 2.063492063492064 |
| b. indicated in case of hospitalization of the patient | 2.7432432 | 2.825396825396826 |
### Chart
| Category | |
|---|---|
### Chart
| Category | |
|---|---|
### Chart
| Category | |
|---|---|
### Chart
| Category | |
|---|---|
Pre
### Chart
| Category | |
|---|---|
### Chart
| Category | |
|---|---|
### Chart
| Category | |
|---|---|
### Chart
| Category | |
|---|---|
Post

## Slide 17
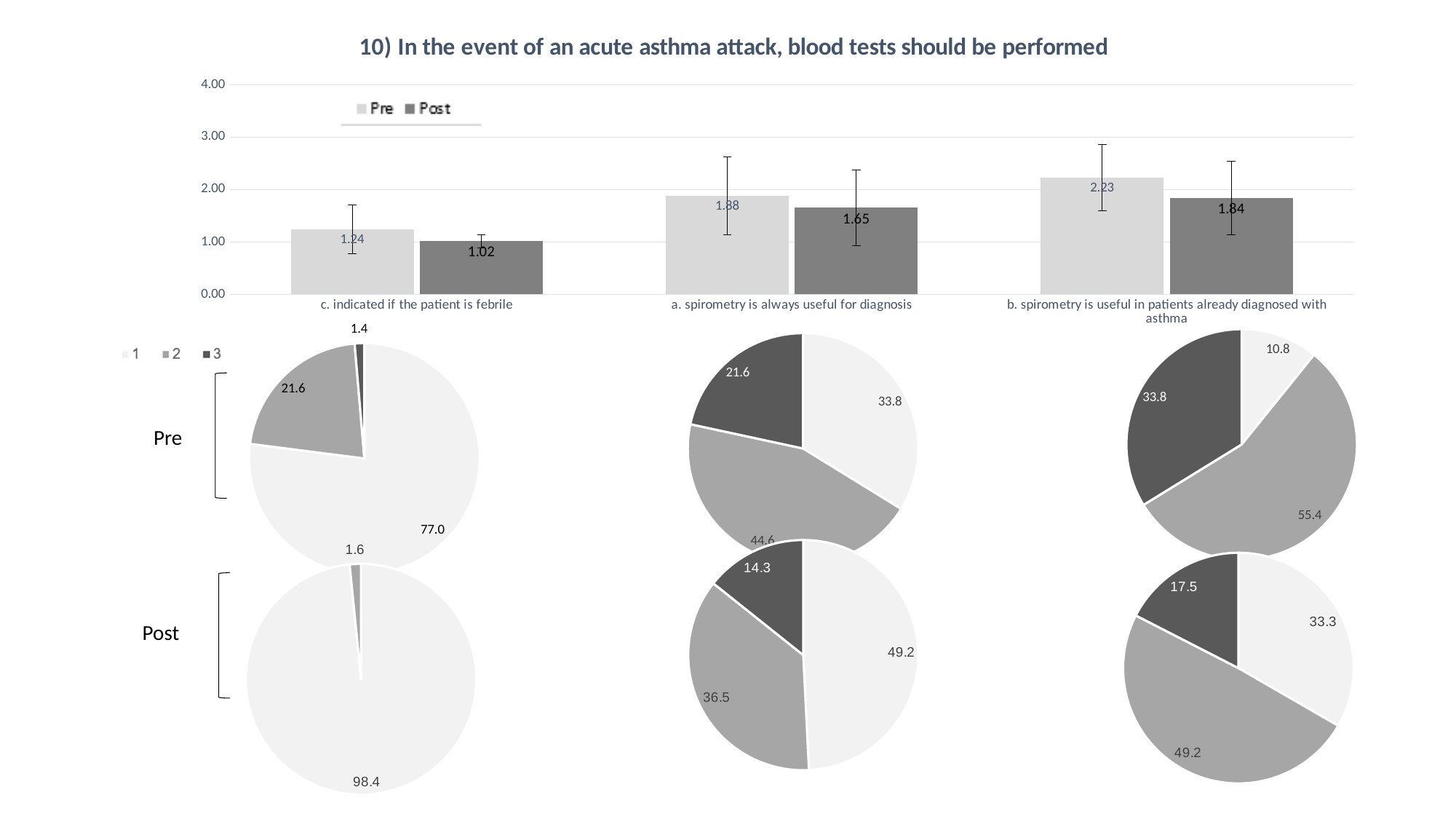

### Chart: 10) In the event of an acute asthma attack, blood tests should be performed
| Category | | |
|---|---|---|
| c. indicated if the patient is febrile | 1.2432432 | 1.015873015873016 |
| a. spirometry is always useful for diagnosis | 1.8783784 | 1.650793650793651 |
| b. spirometry is useful in patients already diagnosed with asthma | 2.2297297 | 1.841269841269841 |
### Chart
| Category | |
|---|---|
### Chart
| Category | |
|---|---|
### Chart
| Category | |
|---|---|
Pre
### Chart
| Category | |
|---|---|
### Chart
| Category | |
|---|---|
### Chart
| Category | |
|---|---|
Post

## Slide 18
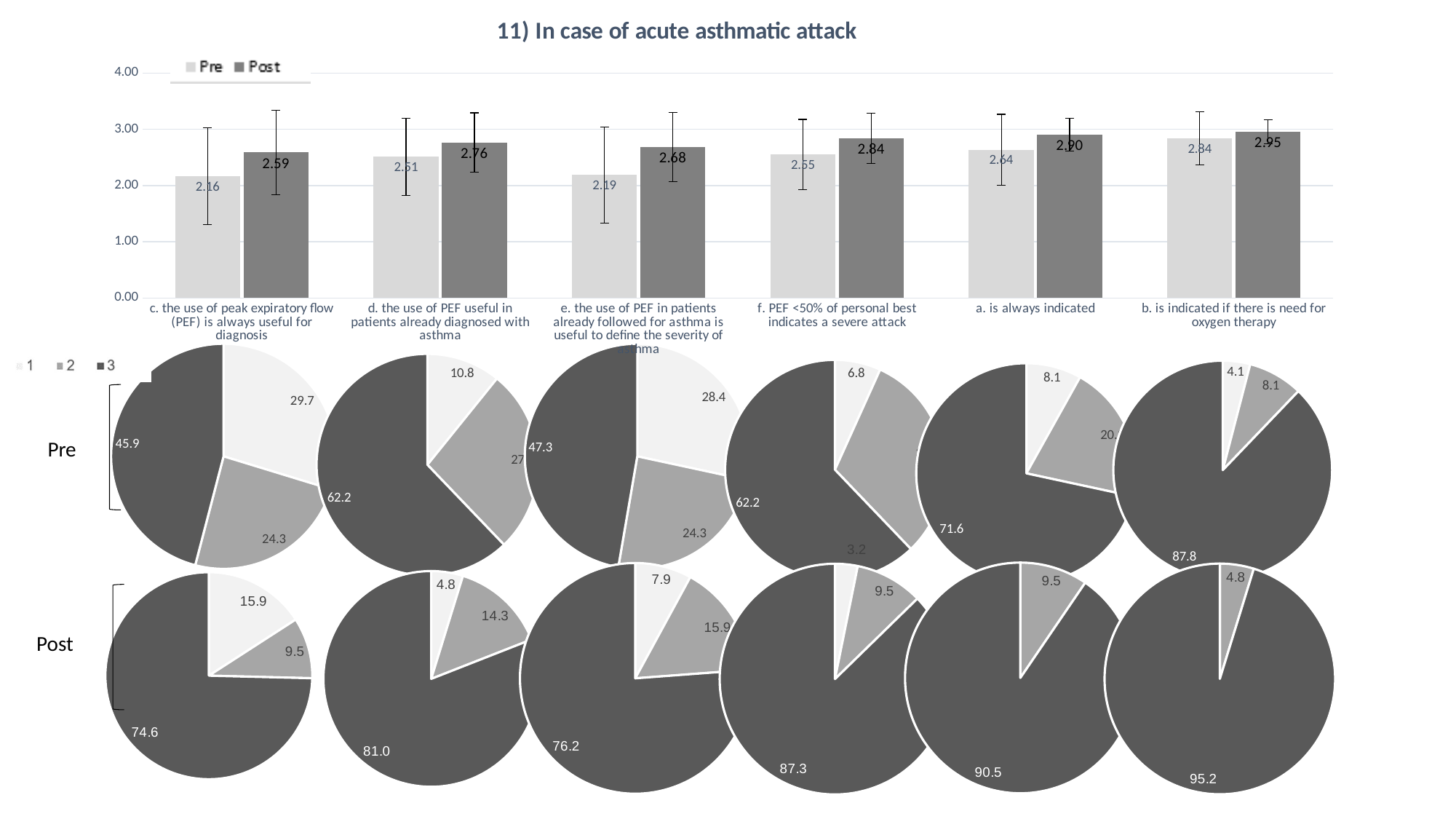

### Chart: 11) In case of acute asthmatic attack
| Category | | |
|---|---|---|
| c. the use of peak expiratory flow (PEF) is always useful for diagnosis | 2.1621622 | 2.587301587301587 |
| d. the use of PEF useful in patients already diagnosed with asthma | 2.5135135 | 2.761904761904762 |
| e. the use of PEF in patients already followed for asthma is useful to define the severity of asthma | 2.1891892 | 2.682539682539682 |
| f. PEF <50% of personal best indicates a severe attack | 2.5540541 | 2.841269841269841 |
| a. is always indicated | 2.6351351 | 2.904761904761905 |
| b. is indicated if there is need for oxygen therapy | 2.8378378 | 2.952380952380952 |
### Chart
| Category | |
|---|---|
### Chart
| Category | |
|---|---|
### Chart
| Category | |
|---|---|
### Chart
| Category | |
|---|---|
### Chart
| Category | |
|---|---|
### Chart
| Category | |
|---|---|
Pre
### Chart
| Category | |
|---|---|
### Chart
| Category | |
|---|---|
### Chart
| Category | |
|---|---|
### Chart
| Category | |
|---|---|
### Chart
| Category | |
|---|---|
### Chart
| Category | |
|---|---|
Post

## Slide 19
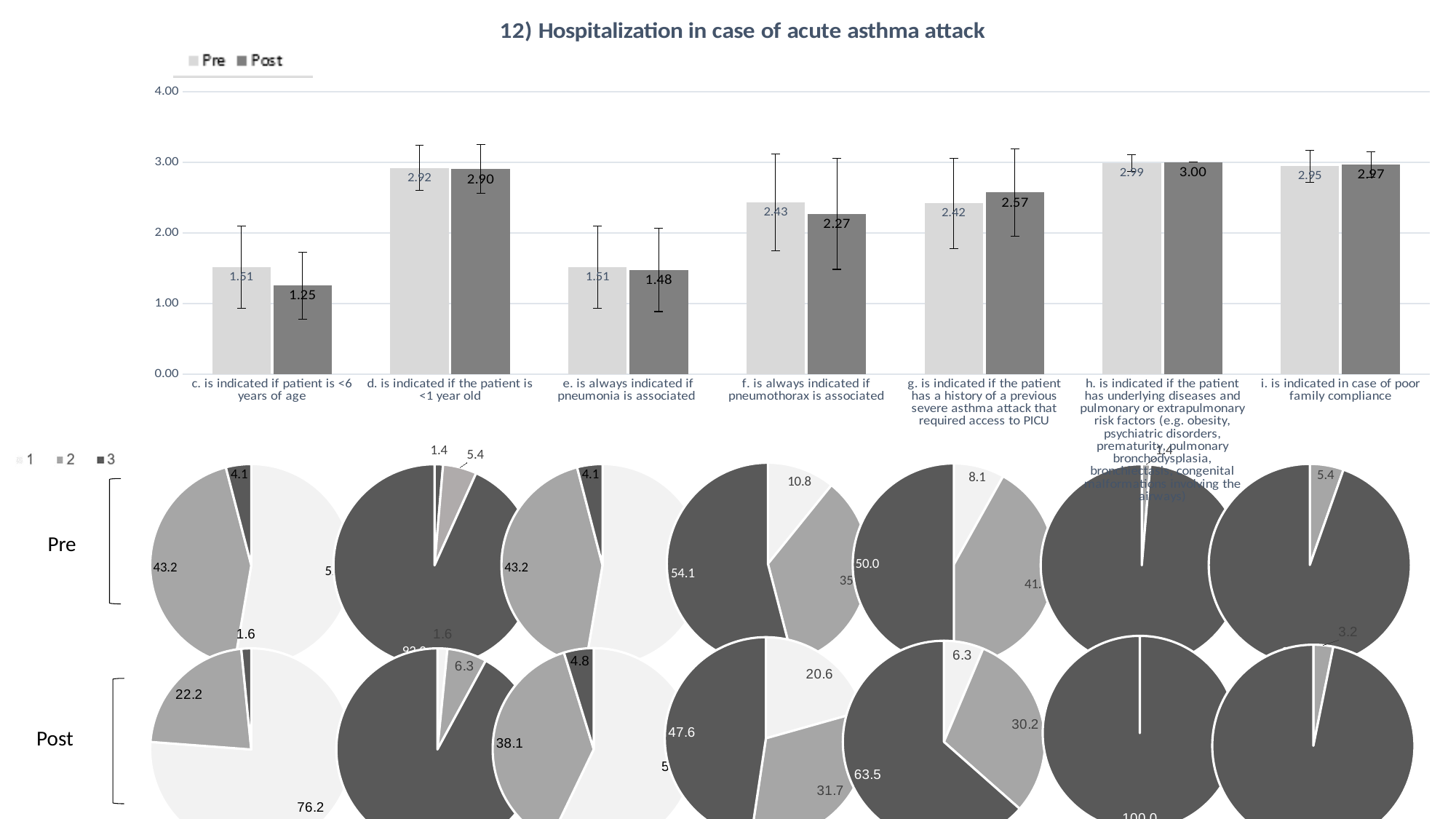

### Chart: 12) Hospitalization in case of acute asthma attack
| Category | | |
|---|---|---|
| c. is indicated if patient is <6 years of age | 1.5135135 | 1.253968253968254 |
| d. is indicated if the patient is <1 year old | 2.9189189 | 2.904761904761905 |
| e. is always indicated if pneumonia is associated | 1.5135135 | 1.476190476190476 |
| f. is always indicated if pneumothorax is associated | 2.4324324 | 2.26984126984127 |
| g. is indicated if the patient has a history of a previous severe asthma attack that required access to PICU | 2.4189189 | 2.571428571428572 |
| h. is indicated if the patient has underlying diseases and pulmonary or extrapulmonary risk factors (e.g. obesity, psychiatric disorders, prematurity, pulmonary bronchodysplasia, bronchiectasis, congenital malformations involving the airways) | 2.9864865 | 3.0 |
| i. is indicated in case of poor family compliance | 2.9459459 | 2.968253968253968 |
### Chart
| Category | |
|---|---|
### Chart
| Category | |
|---|---|
### Chart
| Category | |
|---|---|
### Chart
| Category | |
|---|---|
### Chart
| Category | |
|---|---|
### Chart
| Category | |
|---|---|
### Chart
| Category | |
|---|---|
Pre
### Chart
| Category | |
|---|---|
### Chart
| Category | |
|---|---|
### Chart
| Category | |
|---|---|
### Chart
| Category | |
|---|---|
### Chart
| Category | |
|---|---|
### Chart
| Category | |
|---|---|
### Chart
| Category | |
|---|---|
Post

## Slide 20
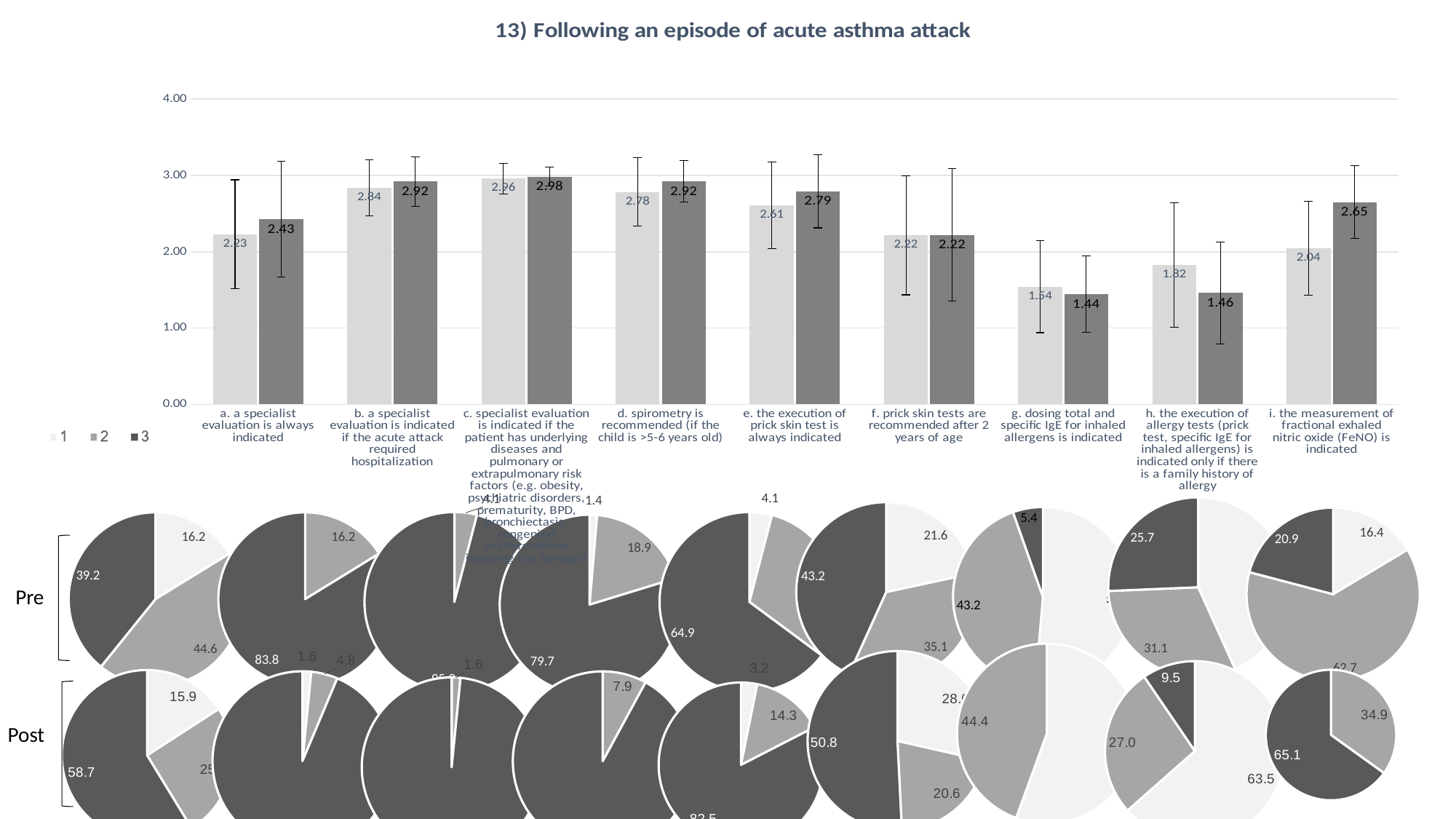

### Chart: 13) Following an episode of acute asthma attack
| Category | | |
|---|---|---|
| a. a specialist evaluation is always indicated | 2.2297297 | 2.428571428571428 |
| b. a specialist evaluation is indicated if the acute attack required hospitalization | 2.8378378 | 2.92063492063492 |
| c. specialist evaluation is indicated if the patient has underlying diseases and pulmonary or extrapulmonary risk factors (e.g. obesity, psychiatric disorders, prematurity, BPD, bronchiectasis, congenital malformations involving the airways) | 2.9594595 | 2.984126984126984 |
| d. spirometry is recommended (if the child is >5-6 years old) | 2.7837838 | 2.92063492063492 |
| e. the execution of prick skin test is always indicated | 2.6081081 | 2.793650793650793 |
| f. prick skin tests are recommended after 2 years of age | 2.2162162 | 2.222222222222222 |
| g. dosing total and specific IgE for inhaled allergens is indicated | 1.5405405 | 1.444444444444444 |
| h. the execution of allergy tests (prick test, specific IgE for inhaled allergens) is indicated only if there is a family history of allergy | 1.8243243 | 1.46031746031746 |
| i. the measurement of fractional exhaled nitric oxide (FeNO) is indicated | 2.0447761 | 2.650793650793651 |
### Chart
| Category | |
|---|---|
### Chart
| Category | |
|---|---|
### Chart
| Category | |
|---|---|
### Chart
| Category | |
|---|---|
### Chart
| Category | |
|---|---|
### Chart
| Category | |
|---|---|
### Chart
| Category | |
|---|---|
### Chart
| Category | |
|---|---|
### Chart
| Category | |
|---|---|
Pre
### Chart
| Category | |
|---|---|
### Chart
| Category | |
|---|---|
### Chart
| Category | |
|---|---|
### Chart
| Category | |
|---|---|
### Chart
| Category | |
|---|---|
### Chart
| Category | |
|---|---|
### Chart
| Category | |
|---|---|
### Chart
| Category | |
|---|---|
### Chart
| Category |
|---|
### Chart
| Category |
|---|
### Chart
| Category |
|---|
### Chart
| Category |
|---|
### Chart
| Category |
|---|
### Chart
| Category | |
|---|---|
Post
